# Nursing workload: use of artificial intelligence to develop a classifier model ^*^

**DOI:** 10.1590/1518-8345.7131.4239

**Published:** 2024-07-05

**Authors:** Ninon Girardon da Rosa, Tiago Andres Vaz, Amália de Fátima Lucena

**Affiliations:** ^1^ Universidade Federal do Rio Grande do Sul, Escola de Enfermagem, Porto Alegre, RS, Brazil.; ^2^ Hospital de Clínicas de Porto Alegre, Diretoria de Enfermagem, Porto Alegre, RS, Brazil.; ^3^ University Medical Center Utrecht, Data Science and Bioestatistic, Utrecht, Netherlands.; ^4^ Hospital de Clínicas de Porto Alegre, Comissão do Processo de Enfermagem, Porto Alegre, RS, Brazil.; ^5^ Scholarship holder at the Conselho Nacional de Desenvolvimento Científico e Tecnológico (CNPq), Brazil.

**Keywords:** Nursing, Workload, Nursing Informatics, Electronic Health Records, Artificial Intelligence, Machine Learning

## Abstract

**Objective:**

to describe the development of a predictive nursing workload classifier model, using artificial intelligence.

**Method:**

retrospective observational study, using secondary sources of electronic patient records, using machine learning. The convenience sample consisted of 43,871 assessments carried out by clinical nurses using the Perroca Patient Classification System, which served as the gold standard, and clinical data from the electronic medical records of 11,774 patients, which constituted the variables. In order to organize the data and carry out the analysis, the Dataiku® data science platform was used. Data analysis occurred in an exploratory, descriptive and predictive manner. The study was approved by the Ethics and Research Committee of the institution where the study was carried out.

**Results:**

the use of artificial intelligence enabled the development of the nursing workload assessment classifier model, identifying the variables that most contributed to its prediction. The algorithm correctly classified 72% of the variables and the area under the Receiver Operating Characteristic curve was 82%.

**Conclusion:**

a predictive model was developed, demonstrating that it is possible to train algorithms with data from the patient’s electronic medical record to predict the nursing workload and that artificial intelligence tools can be effective in automating this activity.

## Highlights

**(1)** Development of a predictive classifier model for nursing workload.

**(2)** Identification of the main variables that generate nursing workload.

**(3)** Possibility of automating the assessment of nursing workload.

**(4)** Qualification of care management.

**(5)** Contribution to personnel sizing studies.

## Introduction

 Nursing workload is characterized by the work demand required of nursing professionals in patient care activities ^(^ ^1^ ^)^ . There are classification systems that categorize patients according to the quantity, complexity and time spent on care. Several classification systems are used around the world, with no scientific basis for one system to have preference over another, but they all aim to estimate the workload, providing parameters for planning the necessary care resources ^(^ ^2^ ^)^ . 

 In Brazil, Perroca’s Patient Classification System (PCS) is used for adult patients hospitalized in inpatient units. Nine areas of care are considered in its assessment: planning and coordination of the care process, investigation and monitoring, body care and eliminations, skin and mucous membrane care, nutrition and hydration, locomotion and activity, therapy, emotional support and health education. Each indicator has a gradation from 1 to 4, indicating the increasing complexity of care. These values combined lead to classification into one of four categories of care: minimum, intermediate, semi-intensive and intensive, which determine the patient’s degree of dependence on nursing care ^(^ ^3^ ^)^ . 

 The continuous assessment of workload has positive implications for managing the nurses’ work process, although it has a degree of subjectivity and requires dedicated time to apply the instruments, amidst so many care and management responsibilities developed ^(^ ^4^ ^)^ . This context demonstrates the importance of improving this process, as workload assessment provides parameters for staffing in Brazilian health institutions ^(^ ^5^ ^)^ . A balanced staff provides essential care for patients, prevention of adverse events, as well as safety for professionals and job satisfaction ^(^ ^2^ ^)^ . 

 In hospitals, the growing investment in Electronic Patient Records (EPR) enables the systematization of data from professionals’ care records and the use of innovative techniques for agile processing of large volumes of clinical data and predictive analysis, in addition to the development of tools that automate activities ^(^ ^6^ ^)^ . 

 Thus, nurses’ approach to data science expands the possibilities of developing innovative solutions, with useful information, extracted from databases ^(^ ^7^ ^)^ , especially in institutions where there is an environment favorable to the computerization of all stages of the Nursing Process (NP), which promotes the systematization of data and the use of standardized language, such as the Nursing Diagnoses (NDs) of NANDA International (NANDA-I) ^(^ ^8^ ^)^ and the interventions and activities of the Classification of Nursing Interventions (NIC) ^(^ ^9^ ^)^ . 

 Analytical databases introduce concepts related to data volumes, speed, variety, veracity and value, which characterize the definition of Big Data, demonstrating identity with this dynamic context of hospitals, which are benefiting from the development of intelligent systems ^(^ ^7^ ^)^ . 

 Artificial Intelligence (AI) demonstrates intelligent behavior and can develop activities with some degree of autonomy to achieve specific objectives, which is why its use in nursing can offer data assertiveness and reduce working time ^(^ ^10^ ^)^ . 

 AI techniques, such as machine learning, may help us answering nursing problems and nurses engaged in this global discussion raise the quality of practice and care management and have a lot to contribute to the development of computerized systems and the development of predictive models ^(^ ^10^ ^)^ . 

 These advances have been driven by multidisciplinary studies with healthcare professionals and data scientists, who seek to develop models that meet the complexity of the hospital environment ^(^ ^7^ ^,^ ^10^ ^-^ ^11^ ^)^ . 

 Although Brazilian hospitals need to face challenges related to technological infrastructure, data systematization and interoperability between systems for the use of AI tools, nurse leadership has a fundamental role in the search for innovations and the development of skills, so that nursing professionals area can be prepared for an increasingly computerized world ^(^ ^11^ ^)^ . 

 The application of AI in nursing care environments is recent and has been explored mainly in hospitals, in image and signal processing, in classifying nursing activities, in communicating care and in detecting falls. The identification of other areas of application of this technique can generate important contributions to teaching, research and nursing practice ^(^ ^12^ ^)^ . 

 Regarding the assessment of nursing workload, in a literature review, no Brazilian studies were found that apply AI to carry out this measurement. Few international publications address the use of automated instruments ^(^ ^13^ ^-^ ^15^ ^)^ , however, there is no mention of the use of data generated by the NP, which is a reference methodology for organizing care and nursing records ^(^ ^16^ ^)^ . 

 The North American Nursing Association (ANA) encourages the development of automated nursing workload assessment systems, highlighting the importance of scientific basis and the ability to integrate with institutional platforms. ANA emphasizes that instruments must be simple and efficient, to translate the reality of each location and not generate additional workload for nurses, but they need to be validated and their results monitored in order to offer correct predictions about the needs of teams and employees. patients ^(^ ^17^ ^)^ . The operationalization of these recommendations depends on the development of joint research between health professionals and data scientists, in order to overcome this knowledge gap and possible weaknesses in the use of secondary data and AI ^(^ ^17^ ^)^ . 

Based on these ideas and the lack of research on the topic, this article aims to describe the development of a predictive nursing workload classifier model, using AI.

## Method

### Study design

 This is a retrospective observational study, using secondary sources of electronic patient records, with the development of a predictive classifier model with supervised Machine Learning (ML) techniques ^(^ ^18^ ^)^ . 

### Study setting

The study took place from 2021 to 2023, in a large public university hospital, providing care for highly complex patients. The institution is internationally accredited in quality and safety standards and its pillars are excellent assistance, teaching and research, and innovation. The hospital developed its own computerized system in the 80s, which has been improved since then. The NP and workload assessment instruments are already incorporated, but they are not interoperable with each other.

### Data logistics

 The researchers used the stages of the Knowledge Discovery in Databases (KDD) ^(^ ^18^ ^)^ process as a roadmap for data logistics: selection, pre-processing, transformation and mining of data and interpretation of results. KDD ^(^ ^19^ ^)^ is a process for identifying consistent patterns in large amounts of data and discovering relevant information to support strategic decisions. 

### Structuring the analytical database

 The structuring of the analytical database involves data selection, pre-processing and transformation ^(^ ^19^ ^)^ . 

 In the selection stage, the study data was defined, which was organized into two sets, followed by data collection and import into the Dataiku ^®^ data science platform, associated with the Big Data processing software PostgreSQL ^®^ , which allowed database management and the relationship of complex data to each other. 

 The first set of data was organized from 107,507 patient assessments carried out by clinical nurses from twelve clinical-surgical inpatient units, from 2015 to 2019, using Perroca’s PCS ^(^ ^3^ ^)^ . These data correspond to patients’ scores and their respective classifications in the categories of minimum, intermediate, semi-intensive and intensive care. The spreadsheets contained the patient’s bed number and the date of evaluation. The data were collected by the researchers directly from computerized spreadsheets in the institution’s system and were organized in a single spreadsheet to be used as a gold standard. 

 The second set was organized based on data extracted from the EPR of patients admitted to the same units, in the same period as the first set of data. The request to consult medical records (query) was planned in meetings with the Information Technology (IT) analyst who carries out this activity at the institution, considering the variables under study. Thus, in each query, a variable representing the nursing workload was requested, based on the nine areas of care of Perroca’s PCS ^(^ ^3^ ^)^ : nursing care, nursing diagnoses, records of vital signs, diet prescriptions, prescription of medications, prescription of solutions, prescription of hemotherapy, prescription of oxygen therapy, requests for exams collected by nursing, requests for consultations by specialists and the existence of educational practices for patients and families. 

In all queries, the variables age, sex, education, date of hospitalization and bed number and medical record identifiers were also requested. Thus, the study included 15 different variables, which were expanded, considering their stratifications, such as: nursing care totaled 846 and nursing diagnoses totaled 189 variables. The data available in tables referred to 58,888 hospitalized patients. Among these, those who were registered in the same bed and on the same date of hospitalization as the patients in the first set of data were selected, which resulted in 11,774 patients.

Data prior to 2015 were not included in the model, as there are no systematized records of patient assessment with the Perroca PCS, and data prior to 2019 was not included, because care processes were changed during the pandemic caused by the coronavirus.

In the pre-processing stage, researchers performed review and cleaning to ensure data quality.

 For the first set of data, the exclusion criteria adopted were: different patients who had evaluations with Perroca’s PCS ^(^ ^3^ ^)^ on the same day and were registered in the same bed, because there was doubt whether the evaluation recorded was from the patient who left or from the patient who entered the bed; patient with two different assessments on the same day and who was registered in the same bed, due to the possibility of error when registering the patient’s bed or the possibility of a second registration relating to the same patient to correct an assessment error, instead of correction have been made on the first registration; patients with less than 48 hours of hospitalization at the time of evaluation, as it was inferred that these patients would be less likely to be exposed to the variables established for the analysis. After applying these criteria to the set of 107,507 evaluations, 43,871 evaluations were obtained for the study. 

In relation to the second set of data, the pre-processing stage required a lot of time and dedication from researchers in the review, however the reorganization of tables did not result in a change in the number of patients, corroborating the 11,774 patients.

 Data from both sets were imported into the Dataiku ^®^ data science platform as they became available. 

In the transformation stage, the raw data was processed to have uniformity and to be mined with AM techniques, in search of patterns and relationships in the large data set (Big Data)

### Data mining

 Data mining is the process of analyzing large sets of data to identify patterns and extract information that generates knowledge and supports decisions ^(^ ^19^ ^)^ . In data mining, LM algorithms are used, which define the sequence of procedures through automated mathematical instructions ^(^ ^18^ ^)^ . This study was developed in the Python language, with the classification algorithms module from the Scikit-learn ^(^ ^20^ ^)^ package. The algorithm applied was Random Forest (RF), characterized by a combination of predictors that are developed in the form of decision trees and distributed across classes according to the degree of purity and homogeneity ^(^ ^21^ ^)^ . 

 In developing the predictive model, the k-fold cross-validation technique (k=5) was used, dividing the analytical database into k partitions for the model to be trained on k-1 partitions and validated on the remaining partition. This procedure was repeated, alternating the partition of the training data ^(^ ^22^ ^)^ , seeking to adjust the patients’ care class (minimum, intermediate, intensive, semi-intensive). 

 The process included strategies to guarantee the quality of the steps: improvements in the parameters of the RF algorithm, which reached a final composition of 1000 trees, six layers deep ^(^ ^21^ ^)^ , and checks to avoid overfitting (excessive adjustment that occurs when the model machine learning provides accurate predictions only for training data, as if the model is not able to generalize to the other unseen data) and data leakage (sharing data between the training and validation phases). These adjustments increased the accuracy to the performance saturation point of the model metrics ^(^ ^22^ ^)^ . 

 The metrics evaluated resulted from the confusion matrix (accuracy, sensitivity, F1 score and precision) and the performance of the ROC curve (Receiver Operating Characteristic) and AUC-ROC (Area Under The ROC Curve) ^(^ ^22^ ^)^ . 

 The importance of each variable for the final classification of nursing workload was known in the model prediction phase ^(^ ^23^ ^)^ . 

### Data analysis

 For data analysis, the Dataiku ^®^ data science platform and the Big Data processing software PostgreSQL ^®^ were used. Exploratory analysis was carried out at all stages of the research to understand the data and the relationships between the variables ^(^ ^19^ ^)^ . Descriptive analysis was used to detail the characteristics of the data sets and the results of the variables were presented in relative frequency. Predictive analysis occurred in the results interpretation phase, making it possible to explore the relationships between variables from previous events and variables predicted in the developed model, and to understand the performance of the corresponding metrics ^(^ ^18^ ^)^ . 

### Ethical aspects

 The study was approved by the institutional Research and Ethics Committee (2021-0521) and registered on *Plataforma Brasil* (55207921.50000.5327). The recommendations of the Data Use Commitment Form were adopted by the researchers and the use of the Informed Consent Form was waived. 

## Results

### Characterization of patient assessments carried out by nurses with Perroca’s PCS, referring to the first set of data

The assessments carried out by nurses comprised 43,871 records. The majority of patients are in the semi-intensive care class (50.61%), followed by the intermediate care class (31.25%). The average age is 61 years old [Standard Deviation (SD)±16.4], 50.08% are male and 52.77% have primary education.

### Characterization of patients from whom data were extracted from electronic medical records to compose the variables of the second set of data

Patients admitted to clinical-surgical units were characterized by 11,774 people, with a mean age of 61.6 years (SD ±16.7), 50.95% were male and 54.39% had primary education.

### Importance of variables in the predictive model for assessing nursing workload

**Figure 1:**
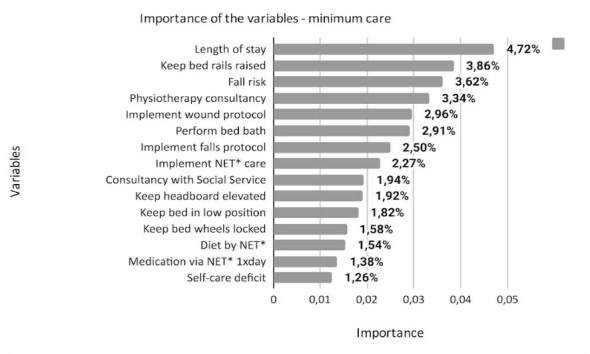
- Description of the importance of the variables in the minimum care class of the nursing workload assessment predictive model (n = 574). Porto Alegre, RS, Brazil, 2023

**Figure 2:**
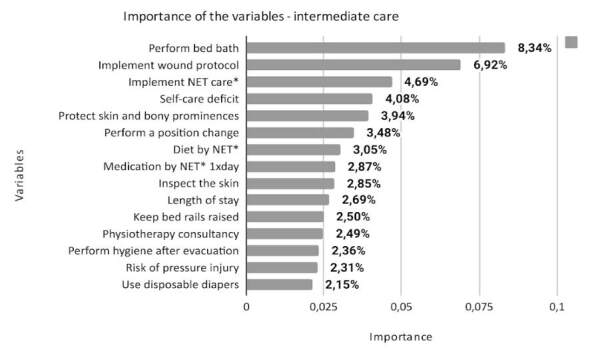
- Description of the importance of variables in the intermediate care class of the nursing workload assessment predictive model (n = 701). Porto Alegre, RS, Brazil, 2023

**Figure 3:**
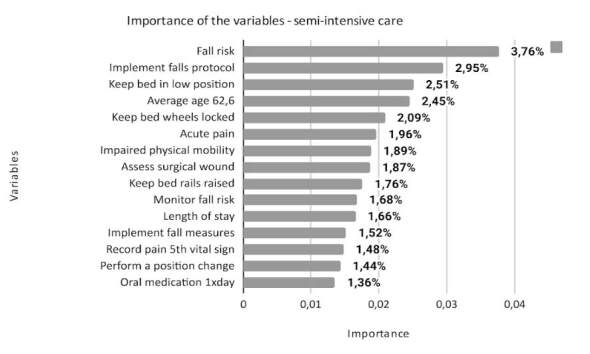
- Description of the importance of variables in the semi-intensive care class of the nursing workload assessment predictive model (n = 731). Porto Alegre, RS, Brazil, 2023

**Figure 4:**
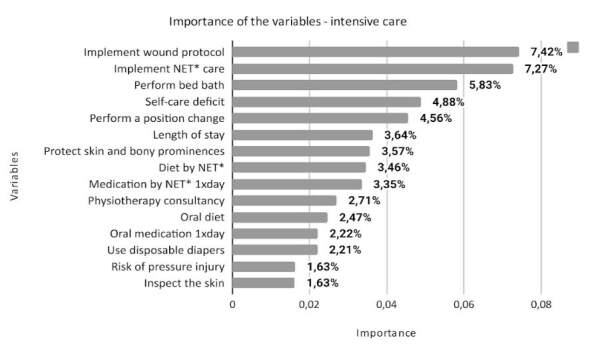
- Description of the importance of variables in the intensive care class in the predictive model for assessing nursing workload (n = 692). Porto Alegre, RS, Brazil, 2023

 The RF algorithm was trained to classify the variables into the four care classes of Perroca’s PCS ^(^ ^3^ ^)^ . The total number of variables that make up each class is different: 574 for minimum, 701 for intermediate, 731 for semi-intensive and 692 for intensive. This article presents the 15 most important variables for each class, according to the degree of importance in the model, as shown in Figures 1 , 2 , 3 and 4 . 

* NET = Nasoenteral Tube

* NET = Nasoenteral Tube

* NET = Nasoenteral Tube

### Classifier model performance evaluation metrics

 Based on the classifications of the variables, the model sought to identify the correct positive predictions. The results of the metrics are shown in Table 1 . 

 All metrics performed excellently in the intermediate care class (0.962), followed by the minimum care class (0.917). This means that the algorithm made a good distinction between the care required for patients in these two classes. The semi-intensive (0.759) and intensive (0.791) care classes had lower results. Models with performance between 0.8 < 0.9 are considered good and those with performance ≥ 0.9 are excellent ^(^ ^21^ ^)^ . 

**Table 1:** - Metrics for evaluating the performance of the predictive classifier model of nursing workload, according to the classification into minimum, intermediate, semi-intensive and intensive care of the PCS ^*^ of Perroca. Porto Alegre, RS, Brazil, 2023

**PCS** ^*^ **Perroca**	**Accuracy**	**Sensitivity**	**Precision**	**F1** **Score** ^†^	**AUC- ROC** ^‡^
Minimum	0,990	0,715	0,849	0,776	0,917
Intermediaries	0,952	0,929	0,917	0,923	0,962
Semi-intensive	0,691	0,965	0,625	0,759	0,759
Intensive	0,824	0,463	0,459	0,461	0,791
Final Model	0,72	0,66	0,67	0,64	0,82

^*^ Perroca`s PCS = Perroca`s Patient Classification System;

^†^ F1 Score = Function 1 Score;

^‡^ AUC-ROC = Area Under The Receiver Operating Characteristic Curve

 The values of the final model are based on the Multiclass Area Under the Curve approach, which classified the four classes at the same time and presented a SD of ± 0.036 ^(^ ^21^ ^-^ ^24^ ^)^ . 

 Overall, the RF algorithm correctly classified 72% of the model’s variables and the AUC-ROC indicated that the model performed 82% in the overall classification of patients in the different care categories ^(^ ^21^ ^)^ . 

## Discussion

 It is observed that the majority of patients evaluated with the Perroca PCS ^(^ ^3^ ^)^ are elderly people, with primary education and are part of the semi-intensive care class. It is also identified that, in the classification of EPR records, the RF algorithm selected similar variables to compose the care classes, however, it attributed different degrees of importance to them. Nursing care was the most significant variable in all classes, reinforcing the researched phenomenon: the classification of nursing workload, which has in its essence the characterization of the work demand required of these professionals in daily patient care activities ^(^ ^1^ ^)^ . 

 In the clinical practice of the institution under study, this prescribed care results from the diagnoses made by nurses, which are based on the NANDA-I standardized language system ^(^ ^8^ ^)^ and are related to the construct of Perroca’s PCS, based on the identification of needs of patient care ^(^ ^3^ ^)^ . Comparative study between the NDs and the care areas of the Perroca PCS, in the same scenario, found correspondence between them in at least one care area of the instrument, demonstrating that the NDs can indicate, in addition to care needs, the degree of complexity of care and dependence on the nursing team, being important guides in care management ^(^ ^25^ ^)^ . 

 Another relevant aspect to be highlighted is the characteristics of the patients in the study, when compared to the definition of each class of care in Perroca’s PCS, as in all of them there are variables specific to patients dependent on nursing, probably due to the predominance of elderly people, which reinforces the importance of nursing care in attention to basic human needs. This framework, recommended in studies on NP in Brazil, also supported Perroca’s PCS, since basic human needs are vital phenomena for human beings and, therefore, defined as concrete entities of Nursing Science ^(^ ^26^ ^-^ ^27^ ^)^ . 

 In the minimum care class, in which, conceptually, patients are stable from a clinical point of view and physically self-sufficient in terms of their basic human needs ^(^ ^3^ ^)^ , the length of stay (period between the date of admission to the unit and the date of its evaluation with Perroca’s PCS) was the most important variable (4.72%), suggesting that, even for patients who theoretically require less care, the increase in hospitalization time can generate workload for the nursing team. 

 It is noteworthy that the social service consultancy variable also stands out, confirming a profile of patients who, in addition to direct nursing care, have problems that require multidisciplinary team approaches, with interventions that facilitate the interface between the health care system with the patient and family. This demands an organization that is directly related to the patient’s length of stay ^(^ ^9^ ^)^ . 

 A North American study, which developed a BF model to improve the hospital discharge process, identified the following as the main potential barriers to discharge: the patient who does not have a regular oral diet, the unavailability of home visiting services and the lack of social support ^(^ ^28^ ^)^ . A European study on causes of delayed hospital discharge refers, among the multiple contributing factors, to difficulties in transitioning care from hospital to home or to long-term care institutions ^(^ ^29^ ^)^ . 

 Other variables emerged in this class of care, but the characteristics of the patients do not reflect Perroca’s definition ^(^ ^3^ ^)^ , because they are diagnoses and nursing care typical of dependent patients and, although they have been classified as belonging to the minimum care class, the variables are repeated in other classes of care. Examples of this are the Nursing Diagnosis (ND) deficit in self-care ^(^ ^8^ ^)^ , indicating inability to carry out routine activities of daily living, such as eating and bathing; nursing care related to the basic physiological ^(^ ^9^ ^)^ (performing bed bath, implementing nasoenteral tube care) and complex physiological ^(^ ^9^ ^)^ (implementing wound treatment prevention protocol) domains; in addition to physiotherapy consultancy. 

 The ND risk of falls ^(^ ^8^ ^)^ , together with care in the patient safety domain ^(^ ^9^ ^)^ (keeping bed rails raised, implementing a fall prevention protocol, keeping the bed in a low position, keeping bed wheels locked) represent safety interventions. protection against the occurrence of harm, which to a lesser or greater extent is part of universal care for hospitalized patients. 

 The intermediate care class consists of stable patients from a clinical and nursing point of view, with partial dependence on nursing actions to meet basic human needs ^(^ ^3^ ^)^ . In this class, the variable that proved to be most important was nursing care to perform bed baths (8.34%), which, associated with the care of performing hygiene after evacuation and using disposable diapers, comprise body hygiene care that requires physical effort to mobilize the patient and change clothes. bed, which can cause fatigue and musculoskeletal injuries, even in cases where professionals perform the activity in pairs ^(^ ^30^ ^)^ . 

 Bed bathing, as it is a basic physiological care ^(^ ^9^ ^)^ , is part of the nursing team’s daily activities and, depending on staffing and environmental infrastructure conditions, overloads the team that carries out this action ^(^ ^30^ ^)^ . 

 Among the other care measures that stood out are complex physiological measures ^(^ ^9^ ^)^ , related to the risk of pressure injuries ^(^ ^8^ ^)^ : implementing a wound prevention and treatment protocol, protecting the skin and bone prominences, changing position and inspecting the skin in search of hyperemia and ischemia ^(^ ^9^ ^)^ . 

 The class of semi-intensive care presupposes care for chronic patients, stable from a clinical point of view, however, with total dependence on nursing actions in terms of meeting basic human needs ^(^ ^3^ ^)^ . 

In the classification carried out by nurses using Perroca’s PCS, 22,106 patients were classified as needing semi-intensive care, around 50% of the first set of data.

 When applying the RF algorithm to the second set of data, the main variable was the risk of falling ^(^ ^8^ ^)^ (3.76%) and various precautions related to it ^(^ ^9^ ^)^ . Preventing falls is the sixth international safety goal, given its high incidence rates, which correspond to two in every five adverse events ^(^ ^31^ ^)^ . The causes of falls in hospitals are multifactorial ^(^ ^32^ ^)^ and are related to the other important variables of this class of care, such as average age of 62.6, the NDs acute pain and impaired physical mobility and nursing care related to the wound operative. These variables refer to the risk factors for limited walking in the postoperative period, which were also found in different studies ^(^ ^32^ ^-^ ^36^ ^)^ . 

 The nature of nursing activities at the bedside justify the concern of professionals in planning and executing fall prevention strategies, applying safety protocols recommended by national and international government bodies, with the allocation of a team dedicated to managing care risks, in addition to studies on the topic ^(^ ^37^ ^)^ . Furthermore, it is known that adequate staffing and training of teams to prevent falls also influence the incidence of this type of event ^(^ ^35^ ^)^ . 

 The intensive care class comprises care for critically ill patients, at imminent risk to their lives, subject to unstable vital signs, who require permanent and specialized nursing care ^(^ ^3^ ^)^ . This definition by Perroca ^(^ ^3^ ^)^ must be relativized in this study, because patients with severe clinical instability do not stay in inpatient units. However, due to the option of presenting the 15 main variables of each class of care, some variables are not explained in this article, such as prescriptions and care related to vital signs, which appear from the 20 ^th^ variable onwards, and which are mentioned in the definition de Perroca ^(^ ^3^ ^)^ . 

 Nursing care, implementing wound prevention and treatment protocol ^(^ ^9^ ^)^ was the most important variable in this class (7.42%) and, together with other complex physiological care ^(^ ^9^ ^)^ , changing position, protecting skin and bone prominences and inspecting the skin for hyperemia and ischemia are related to the risk of pressure injuries ^(^ ^8^ ^)^ . 

 A global study identified that the occurrence of these injuries is one of the most frequent adverse events in hospitalized patients worldwide, with a general prevalence of 12.8% ^(^ ^38^ ^)^ and occurs mainly in dependent patients, with chronic diseases, prolonged hospitalization, with reduced sense of perception and mobility and nutritional changes ^(^ ^39^ ^)^ . These characteristics are based on several variables that stood out in the intensive care class: in the ND self-care deficit syndrome ^(^ ^8^ ^)^ ; in nursing care, implement nasoenteral tube care, perform bed baths and use disposable diapers; in medical prescriptions, administer diet and medications through a nasoenteral tube; and consultation with a physiotherapist. 

 The risk of pressure injuries is generally determined by prediction scales applied by nurses ^(^ ^40^ ^)^ , but there are initiatives to automate this process, as is the case of a Japanese study that developed a predictive BF model to identify the risk of injury early. by pressure, based on records in the electronic medical record ^(^ ^41^ ^)^ . The objective of this model is also to remove the additional workload generated by this activity ^(^ ^41^ ^)^ , in a similar way to that proposed in our study of the nursing workload assessment model. 

 The variables that configure the four classes of the nursing workload assessment model developed refer to a complex care context and include the nine care areas of Perroca’s PCS ^(^ ^3^ ^)^ , however, considering the most important variable of each class some specificities are observed. 

 The only class in which social weaknesses were highlighted was that of minimum care and they are determining factors for the length of stay, being related to the areas of care, emotional support and health education at the Perroca`s PCS ^(^ ^3^ ^)^ , which require a coordination of care between care teams. 

 In the intermediate care class, the variable performing a bed bath demonstrates the strong influence of basic physiological care ^(^ ^9^ ^)^ and the relationship with the area of body care and eliminations ^(^ ^3^ ^)^ . 

 The semi-intensive and intensive classes are guided by complex physiological care ^(^ ^9^ ^)^ (prevention/treatment of falls and pressure injuries), associated with the areas of locomotion and activity and skin and mucous membrane care ^(^ ^3^ ^)^ . Both require permanent direct and indirect patient care interventions, which require the organization of the care environment and multidisciplinary collaboration ^(^ ^3^ ^)^ . 

 This theoretical model for evaluating nursing workload portrays the reality of a highly complex university hospital, which may justify the particularities of the results found. The uniqueness of each care scenario must be considered to obtain reliable models, knowing that, although algorithms can produce useful predictions and recommendations, human knowledge and experience are essential for decision making ^(^ ^6^ ^)^ . 

This study has limitations related to generalizability, because it uses data from patients from a single institution. Considering that knowledge about machine learning applications designed for nursing workload assessment is scarce, it is suggested to invest in data modeling to improve model performance, especially in semi-intensive and intensive care classes, with the aim of both of them reaching the minimum of 80% correct classifications to be considered with good performance. To this end, it will be important to validate the model developed by collecting electronic data directly from the workload assessment tool (Perroca`s PCS), which was computerized in the institutional system during this study, providing qualification of the standard data gold. Another suggestion is to test the model with the exclusive use of variables with standardized terminologies, fundamental for research involving data science, such as the taxonomies of diagnoses and nursing care (NANDA-I and NIC), which could increase the capacity to search for patterns in the analytical database and to understand the classifications made by the model.

It is noteworthy that replication of the study requires an institutional technological structure, availability of data science platforms and professionals with knowledge in this area, in addition to computerized EP, using a standardized language system. It is also important to highlight that the nursing workload classification models, despite being excellent guidelines for care management, will never represent the totality of patients’ needs as they are countless, multidimensional and interdependent.

It is believed that the classifier model developed adds advances to scientific knowledge in nursing, considering that it is the first Brazilian study to evaluate nursing workload, using AI techniques, with EPR data and using a PCS validated as a gold standard. The study is innovative, as this knowledge is incipient in Nursing, and encourages the automation of processes, qualifying care management.

## Conclusion

In this study, a predictive classifier model of nursing workload was developed, using AI. The result of the model demonstrated that it is possible to train algorithms with EPR data to predict nursing workload. The variables length of stay, bed bath, risk of falling and implementing wound prevention and treatment protocol represented the greatest workload in the final classification of the model, providing visibility to relevant aspects to be considered when planning the scales of activities of the hospital, nursing team and staffing in the studied context. The development of predictive classifier models for nursing workload implies the possibility of automating this activity, making it objective, precise and allowing nurses to allocate the time dedicated to this assessment to other care and management demands. The predictive model has the potential to be applied in different environments, as long as the algorithms are trained with qualified data sets that represent the reality of each location.
