# Supplementary material for: Nursing workload: use of artificial intelligence to develop a classifier model
Source: Rev Lat Am Enfermagem. 2024 Jul 5;32:e4239. doi: 10.1590/1518-8345.7131.4239 (PMC11251687; doi:10.1590/1518-8345.7131.4239)
Supplement: Translation

# Translation: Carga de trabajo de enfermería: uso de inteligencia artificial para el desarrollo de modelo clasificador ^*^

**DOI:** 10.1590/1518-8345.7131.4238

**Authors:** Ninon Girardon da Rosa, Tiago Andres Vaz, Amália de Fátima Lucena

**Affiliations:** ^1^ Universidade Federal do Rio Grande do Sul, Escola de Enfermagem, Porto Alegre, RS, Brasil.; ^2^ Hospital de Clínicas de Porto Alegre, Diretoria de Enfermagem, Porto Alegre, RS, Brasil.; ^3^ University Medical Center Utrecht, Data Science and Bioestatistic, Utrecht, Países Bajos.; ^4^ Hospital de Clínicas de Porto Alegre, Comissão do Processo de Enfermagem, Porto Alegre, RS, Brasil.; ^5^ Becaria del Conselho Nacional de Desenvolvimento Científico e Tecnológico (CNPq), Brasil.

**Keywords:** Enfermería, Carga de Trabajo, Informática Aplicada a la Enfermería, Expedientes Electrónicos de Salud, Inteligencia Artificial, Aprendizaje Automático

## Abstract

**Objetivo:**

describir el desarrollo de un modelo clasificador predictivo de la carga de trabajo de enfermería, utilizando inteligencia artificial.

**Método:**

estudio observacional retrospectivo, en fuentes secundarias de registros electrónicos de pacientes, con uso de aprendizaje automático. La muestra por conveniencia se constituyó de 43.871 evaluaciones realizadas por enfermeras asistenciales con el Sistema de Clasificación de Pacientes de Perroca, que sirvieron como patrón oro, y datos clínicos del expediente electrónico de 11.774 pacientes, que constituyeron las variables. Para la organización de los datos y la realización de los análisis se utilizó la plataforma de ciencia de datos Dataiku ^®^ . El análisis de los datos ocurrió de forma exploratoria, descriptiva y predictiva. Estudio aprobado por el Comité de Ética e Investigación de la institución campo del estudio.

**Resultados:**

el uso de inteligencia artificial posibilitó el desarrollo del modelo clasificador de evaluación de la carga de trabajo de enfermería, identificando las variables que más contribuyeron para su predicción. El algoritmo clasificó correctamente el 72% de las variables y el área bajo la curva *Receiver Operating Characteristic* fue del 82%.

**Conclusión:**

hubo el desarrollo de un modelo predictivo, demostrando que es posible entrenar algoritmos con datos del expediente electrónico del paciente para predecir la carga de trabajo de enfermería y que las herramientas de inteligencia artificial pueden ser efectivas para la automatización de esta actividad.

## Destacados

**(1)** Desarrollo de modelo clasificador predictivo de la carga de trabajo de enfermería.

**(2)** Identificación de las principales variables que generan carga de trabajo de enfermería.

**(3)** Posibilidad de automatización de la evaluación de la carga de trabajo de enfermería.

**(4)** Calificación de la gestión asistencial.

**(5)** Contribución en los estudios de dimensionamiento de personal.

## Introducción

 La carga de trabajo en enfermería se caracteriza por la demanda de trabajo requerida de los profesionales de enfermería en las actividades de cuidado al paciente ^(^ ^1^ ^)^ . Existen sistemas de clasificación que categorizan a los pacientes de acuerdo con la cantidad, complejidad y tiempo dedicado para el cuidado. Varios sistemas de clasificación se utilizan en el mundo, no existiendo una base científica para que un sistema tenga preferencia sobre otro, pero todos buscan estimar la carga de trabajo, proporcionando parámetros para la planificación de los recursos asistenciales necesarios ^(^ ^2^ ^)^ . 

 En Brasil, el Sistema de Clasificación de Pacientes (SCP) de Perroca se utiliza para pacientes adultos hospitalizados en unidades de internación. Se consideran nueve áreas de cuidado en su evaluación: planificación y coordinación del proceso de cuidar, investigación y monitoreo, cuidado corporal y eliminaciones, cuidados con piel y mucosas, nutrición e hidratación, locomoción y actividad, terapéutica, soporte emocional y educación a la salud. Cada indicador posee una graduación de 1 a 4, indicando la complejidad creciente del cuidado. Estos valores sumados conducen a la clasificación en una de las cuatro categorías de cuidados: mínimos, intermedios, semi-intensivos e intensivos, que determinan el grado de dependencia del paciente respecto a los cuidados de enfermería ^(^ ^3^ ^)^ . 

 La evaluación continua de la carga de trabajo trae implicaciones positivas al manejo del proceso de trabajo de los enfermeros, aunque tenga un grado de subjetividad y exija tiempo dedicado para la aplicación de los instrumentos, en medio de tantas asignaciones asistenciales y gerenciales desarrolladas ^(^ ^4^ ^)^ . Este contexto demuestra la importancia del perfeccionamiento de este proceso, pues la evaluación de la carga de trabajo proporciona parámetros para el dimensionamiento de personal de las instituciones brasileñas de salud ^(^ ^5^ ^)^ . Un cuadro de personal equilibrado permite la realización de cuidados esenciales a los pacientes, la prevención de eventos adversos, además de seguridad a los profesionales y satisfacción en el trabajo ^(^ ^2^ ^)^ . 

 En los hospitales, la inversión creciente en el Prontuario Electrónico del Paciente (PEP) permite la sistematización de los datos provenientes de los registros de los atendimientos de los profesionales y la utilización de técnicas innovadoras de procesamiento ágil de grandes volúmenes de datos clínicos y análisis predictivos, además del desarrollo de herramientas que automatizan actividades ^(^ ^6^ ^)^ . 

 Así, la aproximación de los enfermeros con la ciencia de datos amplía las posibilidades de desarrollar soluciones innovadoras, con información útil, extraída de los bancos de datos ^(^ ^7^ ^)^ , principalmente en las instituciones donde hay un ambiente favorable a la informatización de todas las etapas del Proceso de Enfermería (PE), lo que promueve la sistematización de datos y uso de lenguaje estandarizado, tales como los Diagnósticos de Enfermería (DEs) de NANDA Internacional (NANDA-I) ^(^ ^8^ ^)^ y las intervenciones y actividades de la Clasificación de las Intervenciones de Enfermería (NIC) ^(^ ^9^ ^)^ . 

 Los bancos de datos analíticos introducen los conceptos relacionados a volúmenes de datos, velocidad, variedad, veracidad y valor, que caracterizan la definición de *Big Dat* a, demostrando identidad con este contexto dinámico de los hospitales, que se están beneficiando del desarrollo de sistemas inteligentes ^(^ ^7^ ^)^ . 

 La Inteligencia Artificial (IA) demuestra comportamiento inteligente y puede desarrollar actividades con algún grado de autonomía para alcanzar objetivos específicos, por ello su uso en enfermería puede ofrecer asertividad de datos y reducción en el tiempo de trabajo ^(^ ^10^ ^)^ . 

 Las técnicas de IA, como el aprendizaje automático, asisten en las respuestas a los problemas de enfermería y los enfermeros comprometidos en esta discusión mundial elevan la calidad de la práctica y de la gestión asistencial y tienen mucho que contribuir en el desarrollo de sistemas informatizados y desarrollo de modelos predictivos ^(^ ^10^ ^)^ . 

 Estos avances han sido impulsados por estudios multidisciplinarios con profesionales de la salud y científicos de datos, que buscan desarrollar modelos que atiendan a la complejidad del ambiente hospitalario ^(^ ^7^ ^,^ ^10^ ^-^ ^11^ ^)^ . 

 Aunque los hospitales brasileños necesitan enfrentar desafíos relacionados con la infraestructura tecnológica, sistematización de datos e interoperabilidad entre sistemas para la utilización de las herramientas de IA, el liderazgo de los enfermeros tiene un papel fundamental en la búsqueda de innovaciones y en el desarrollo de competencias, para que los profesionales del área puedan estar preparados para un mundo cada vez más informatizado ^(^ ^11^ ^)^ . 

 La aplicación de la IA en ambientes de cuidado de enfermería es reciente y ha sido explorada principalmente en los hospitales, en procesamiento de imágenes y señales, en clasificación de actividades de enfermería, en la comunicación de cuidados y en la detección de caídas. La identificación de otras áreas de aplicación de esta técnica puede generar importantes contribuciones para la enseñanza, para la investigación y para la práctica de enfermería ^(^ ^12^ ^)^ . 

 En lo que respecta a la evaluación de carga de trabajo de enfermería, en revisión de la literatura, no se encontraron estudios brasileños que apliquen IA para la realización de esta medición. Escasas publicaciones internacionales tratan la utilización de instrumentos automatizados ^(^ ^13^ ^-^ ^15^ ^)^ , sin embargo, no hay mención sobre el uso de datos generados por el PE, que es una metodología de referencia para la organización de la asistencia y de los registros de enfermería ^(^ ^16^ ^)^ . 

 La Asociación Norteamericana de Enfermería (ANA) estimula el desarrollo de sistemas de evaluación de carga de trabajo de enfermería automatizados, destacando la importancia del fundamento científico y de la capacidad de integración con las plataformas institucionales. La ANA resalta que los instrumentos deben ser simples y eficientes, para traducir la realidad de cada local y no generar carga de trabajo adicional a los enfermeros, pero necesitan ser validados y sus resultados monitoreados a fin de ofrecer predicciones correctas sobre las necesidades de los equipos y de los pacientes ^(^ ^17^ ^)^ . La operacionalización de estas recomendaciones depende del desarrollo de investigaciones conjuntas entre profesionales de la salud y científicos de datos, de manera a superar esta brecha de conocimientos y las eventuales debilidades en el uso de datos secundarios y IA ^(^ ^17^ ^)^ . 

Basándose en estas ideas y en la carencia de investigaciones sobre el tema, este artículo tiene como objetivo describir el desarrollo de un modelo clasificador predictivo de la carga de trabajo de enfermería, utilizando IA.

## Método

### Tipo de estudio

 Se trata de un estudio observacional retrospectivo, en fuentes secundarias de registros electrónicos de pacientes, con la elaboración de un modelo clasificador predictivo con técnicas supervisadas de Aprendizaje Automático (AA) ^(^ ^18^ ^)^ . 

### Escenario del estudio

El estudio se llevó a cabo de 2021 a 2023, en un hospital público universitario de gran tamaño, con atención a pacientes de alta complejidad. La institución está acreditada internacionalmente en estándares de calidad y seguridad y tiene como pilares la asistencia, la enseñanza y la investigación de excelencia e innovación. El hospital desarrolló su propio sistema informatizado en la década de los 80, el cual ha sido mejorado desde entonces. El PE y los instrumentos de evaluación de carga de trabajo ya están incorporados, pero no poseen interoperabilidad entre sí.

### Logística de los datos

 Los investigadores utilizaron las etapas del proceso de *Knowledge Discovery in Databases* (KDD) ^(^ ^18^ ^)^ como guía en la logística de los datos: selección, pre-procesamiento, transformación y minería de datos e interpretación de resultados. El KDD ^(^ ^19^ ^)^ es un proceso para identificar patrones consistentes en grandes cantidades de datos y descubrir información relevante para apoyar decisiones estratégicas. 

### Estructuración de la base de datos analítica

 La estructuración de la base de datos analítica implica selección, pre-procesamiento y transformación de los datos ^(^ ^19^ ^)^ . 

 En la etapa de selección se definió los datos del estudio, que fueron organizados en dos conjuntos, seguido de la recolección de datos y la importación de los mismos a la plataforma de ciencia de datos Dataiku ^®^ , asociada al *software* de procesamiento de *Big Data* PostgreSQL ^®^ , que permitió el manejo de la base de datos y la relación de datos complejos entre sí. 

 El primer conjunto de datos se organizó a partir de 107.507 evaluaciones de pacientes realizadas por enfermeras asistenciales de doce unidades de internación clínico-quirúrgicas, en el período de 2015 a 2019, utilizando el SCP de Perroca ^(^ ^3^ ^)^ . Estos datos corresponden a los puntajes de los pacientes y las respectivas clasificaciones en las categorías de cuidados mínimos, intermedios, semi-intensivos e intensivos. Las hojas de cálculo contenían el número de la cama del paciente y la fecha de la evaluación. Los datos fueron recolectados por los investigadores directamente de las hojas de cálculo informatizadas del sistema de la institución y fueron organizados en una sola hoja de cálculo para ser usados como patrón oro. 

 El segundo conjunto se organizó a partir de datos extraídos del PEP de pacientes internados en las mismas unidades, en el mismo período que el primer conjunto de datos. La solicitud de consulta a los expedientes ( *query* ) fue planificada en reuniones con el analista de Tecnología de la Información (TI) que realiza esa actividad en la institución, considerando las variables en estudio. Así, en cada *query* se solicitó una variable representativa de la carga de trabajo de enfermería, basada en las nueve áreas de cuidado del SCP de Perroca ^(^ ^3^ ^)^ : cuidados de enfermería, diagnósticos de enfermería, registros de los signos vitales, prescripciones de dieta, prescripción de medicamentos, prescripción de soluciones, prescripción de hemoterapia, prescripción de oxigenoterapia, solicitudes de exámenes recogidos por la enfermería, solicitudes de consultorías por especialistas y existencia de conductas educativas a pacientes y familiares. 

 En todas las *queries* también se solicitaron las variables edad, sexo, escolaridad, fecha de internación y los identificadores número de la cama y del expediente. Así, el estudio contempló 15 diferentes variables, que se ampliaron, considerando sus estratificaciones, como por ejemplo: los cuidados de enfermería totalizaron 846 y los diagnósticos de enfermería totalizaron 189 variables. Los datos disponibles en tablas fueron referentes a 58.888 pacientes internados. Entre estos, se seleccionaron aquellos que estaban registrados en la misma cama y en la misma fecha de internación que los pacientes del primer conjunto de datos, lo que resultó en 11.774 pacientes. 

No se incluyeron en el modelo datos anteriores a 2015, por no haber registros sistematizados de evaluación de los pacientes con el SCP de Perroca y datos posteriores a 2019, porque los procesos asistenciales fueron alterados durante la pandemia causada por el coronavirus.

En la etapa de preprocesamiento, los investigadores realizaron revisión y limpieza para garantizar la calidad de los datos.

 Para el primer conjunto de datos, los criterios de exclusión adoptados fueron: pacientes diferentes que tenían evaluaciones con SCP de Perroca ^(^ ^3^ ^)^ el mismo día y estaban registrados en la misma cama, porque hubo duda si la evaluación registrada era del paciente que salió o del que entró en la cama; paciente con dos evaluaciones diferentes el mismo día y que estaba registrado en la misma cama, debido a la posibilidad de error al registrar la cama del paciente o por la posibilidad de ser un segundo registro relativo al mismo paciente para corregir un error de evaluación, en lugar de la corrección haber sido realizada en el primer registro; pacientes con menos de 48 horas de internación en el momento de la evaluación, pues se infería que estos pacientes tendrían menos chances de exponerse a las variables establecidas para el análisis. Tras aplicar estos criterios en el conjunto de 107.507 evaluaciones se obtuvieron 43.871 evaluaciones para el estudio. 

En relación con el segundo conjunto de datos, la etapa de preprocesamiento exigió bastante tiempo y dedicación de los investigadores en la revisión, sin embargo, la reorganización de tablas no implicó en alteración del número de pacientes, corroborando los 11.774 pacientes.

 Los datos de los dos conjuntos fueron importados a la plataforma de ciencia de datos Dataiku ^®^ a medida que fueron siendo disponibles. 

 En la etapa de transformación, los datos brutos fueron procesados para tener uniformidad y ser minados con técnicas de AM, en busca de patrones y relaciones en el gran conjunto de datos ( *Big Data* ). 

### Minería de los datos

 La minería de datos es el proceso de analizar grandes conjuntos de datos para identificar patrones y extraer información que genera conocimientos y apoya decisiones ^(^ ^19^ ^)^ . En la minería de datos se usan algoritmos de AM, que definen la secuencia de los procedimientos por medio de instrucciones matemáticas automatizadas ^(^ ^18^ ^)^ . Este estudio se desarrolló en el lenguaje *Python* , con el módulo de algoritmos de clasificación del paquete *Scikit-learn* ^(^ ^20^ ^)^ . El algoritmo aplicado fue el *Random Forest* (RF), caracterizado por una combinación de predictores que se desarrollan en forma de árboles de decisión y se distribuyen en las clases conforme el grado de pureza y de homogeneidad ^(^ ^21^ ^)^ . 

 En el desarrollo del modelo predictivo se utilizó la técnica de validación cruzada *k-fold* (k=5), con división de la base de datos analítica en k particiones para que el modelo sea entrenado en k-1 particiones y validado en la partición restante. Este procedimiento fue repetido, alternando la partición de los datos de entrenamiento ^(^ ^22^ ^)^ , buscando acertar la clase de cuidados de los pacientes (mínimo, intermedio, intensivo, semi-intensivo). 

 El proceso incluyó estrategias para garantizar la calidad de las etapas: mejoras en los parámetros del algoritmo RF, los cuales alcanzaron una composición final de 1000 árboles, con seis capas de profundidad ^(^ ^21^ ^)^ , y verificaciones para evitar el *overfitting* (ajuste excesivo que ocurre cuando el modelo de aprendizaje de máquina proporciona predicciones precisas solo para datos de entrenamiento, como si el modelo no fuera capaz de generalizar para otros datos no vistos) y el data *leakage* (compartimiento de datos entre las fases de entrenamiento y validación). Estos ajustes elevaron la precisión hasta el punto de saturación de desempeño de las métricas del modelo ^(^ ^22^ ^)^ . 

 Las métricas evaluadas resultaron de la matriz de confusión (precisión, sensibilidad, F1 score y precisión) y del desempeño de la curva ROC ( *Receiver Operating Characteristic* ) y de la AUC-ROC ( *Area Under The ROC Curve* ) ^(^ ^22^ ^)^ . 

 La importancia de cada variable para la clasificación final de la carga de trabajo de enfermería fue conocida en la fase de predicción del modelo ^(^ ^23^ ^)^ . 

### Análisis de los datos

 Para el análisis de datos se utilizó la plataforma de ciencia de datos Dataiku ^®^ y el *software* de procesamiento de *Big Data* PostgreSQL ^®^ . El análisis exploratorio se realizó en todas las etapas de la investigación para la comprensión de los datos y de las relaciones entre las variables ^(^ ^19^ ^)^ . El análisis descriptivo se utilizó para detallar las características de los conjuntos de datos y los resultados de las variables se presentaron en frecuencia relativa. El análisis predictivo ocurrió en la fase de interpretación de los resultados, permitiendo explorar las relaciones entre variables de eventos anteriores y variables previstas en el modelo desarrollado, y conocer el desempeño de las métricas correspondientes ^(^ ^18^ ^)^ . 

### Aspectos éticos

 El estudio fue aprobado por el Comité de Ética e Investigación institucional (2021-0521) y registrado en la *Plataforma Brasil* (55207921.50000.5327). Se adoptaron las recomendaciones del Término de Compromiso de Uso de Datos por parte de los investigadores y se dispensó el uso del Término de Consentimiento Informado. 

## Resultados

### Caracterización de las evaluaciones de los pacientes realizadas por enfermeras con el SCP de Perroca, referente al primer conjunto de datos

Las evaluaciones realizadas por las enfermeras comprendieron 43.871 registros. La mayoría de los pacientes está en la clase de cuidados semi-intensivos (50,61%), seguida de la clase de cuidados intermedios (31,25%). La edad promedio es de 61 años [Desviación Estándar (DE)±16,4], el 50,08% son de sexo masculino y el 52,77% poseen educación primaria.

### Caracterización de los pacientes de quienes se extrajeron datos de los expedientes electrónicos para componer las variables del segundo conjunto de datos

Los pacientes internados en las unidades clínico-quirúrgicas se caracterizaron por 11.774 personas, con una edad promedio de 61,6 años (DE ±16,7), el 50,95% son de sexo masculino y el 54,39% tienen educación primaria.

### Importancia de las variables en el modelo predictor de evaluación de la carga de trabajo de enfermería

 El algoritmo RF fue entrenado para clasificar las variables en las cuatro clases de cuidados del SCP de Perroca ^(^ ^3^ ^)^ . El número total de variables que componen cada clase es diferente: 574 para mínimos, 701 para intermedios, 731 para semi-intensivos y 692 para intensivos. En este artículo se presentan las 15 variables más importantes para cada clase, según el grado de importancia en el modelo, conforme a las Figuras 1 , 2 , 3 y 4 . 

**Figura 1:** - Descripción de la importancia de las variables en la clase de cuidados mínimos del modelo predictor de evaluación de la carga de trabajo de enfermería (n = 574). Porto Alegre, RS, Brasil, 2023

*SNE = Sonda Nasoenteral

**Figura 2:** - Descripción de la importancia de las variables en la clase de cuidados intermedios del modelo predictor de evaluación de la carga de trabajo de enfermería (n = 701). Porto Alegre, RS, Brasil, 2023

*SNE = Sonda Nasoenteral

**Figura 3:** - Descripción de la importancia de las variables en la clase de cuidados semi-intensivos del modelo predictor de evaluación de la carga de trabajo de enfermería (n = 731). Porto Alegre, RS, Brasil, 2023

**Figura 4:** - Descripción de la importancia de las variables en la clase de cuidados intensivos del modelo predictor de evaluación de la carga de trabajo de enfermería (n = 692). Porto Alegre, RS, Brasil, 2023

^*^ SNE = Sonda Nasoenteral

### Métricas de evaluación del desempeño del modelo clasificador

 A partir de las clasificaciones de las variables, el modelo buscó identificar las predicciones positivas correctas. Los resultados de las métricas están demostrados en la Tabla 1 . 

 Todas las métricas tuvieron excelentes desempeños en la clase de cuidados intermedios (0,962), seguida de la clase de cuidados mínimos (0,917). Esto significa que el algoritmo hizo una buena distinción de los cuidados requeridos para los pacientes de estas dos clases. Las clases de cuidados semi-intensivos (0,759) e intensivos (0,791) tuvieron menores resultados. Los modelos con desempeño entre 0,8 < 0,9 se consideran buenos y aquellos con desempeño ≥ 0,9 son excelentes ^(^ ^21^ ^)^ . 

**Tabla 1:** - Métricas de evaluación del desempeño del modelo clasificador predictivo de la carga de trabajo de enfermería, según la clasificación en cuidados mínimos, intermedios, semi-intensivos e intensivos del SCP ^*^ de Perroca. Porto Alegre, RS, Brasil, 2023

**SCP** ^*^ **Perroca**	**Exactitud**	**Sensibilidad**	**Precisión**	**F1** **Score** ^†^	**AUC- ROC** ^‡^
Mínimos	0,990	0,715	0,849	0,776	0,917
Intermedios	0,952	0,929	0,917	0,923	0,962
Semi-intensivos	0,691	0,965	0,625	0,759	0,759
Intensivos	0,824	0,463	0,459	0,461	0,791
Modelo final	0,72	0,66	0,67	0,64	0,82

^*^ SCP Perroca = Sistema de Clasificación de Pacientes de Perroca;

^†^ F1 Score = *Function 1 Score* ;

^‡^ AUC-ROC = *Area Under The Receiver Operating Characteristic Curve*

 Los valores del modelo final están basados en el enfoque *Multiclass Area Under the Curve* , que clasificó las cuatro clases al mismo tiempo y presentó una DE de ± 0,036 ^(^ ^21^ ^-^ ^24^ ^)^ . 

 De forma global, el algoritmo RF clasificó correctamente el 72% de las variables del modelo y la AUC-ROC indicó que el modelo tuvo un desempeño del 82% en la clasificación general de los pacientes en las diferentes categorías de cuidados ^(^ ^21^ ^)^ . 

## Discusión

 Se observa que la mayoría de los pacientes evaluados con el SCP de Perroca ^(^ ^3^ ^)^ son personas mayores, con educación primaria y pertenecen a la clase de cuidados semi-intensivos. También se identifica que, en la clasificación de los registros del PEP, el algoritmo RF seleccionó variables similares para componer las clases de cuidados, sin embargo, les atribuyó diferentes grados de importancia. Los cuidados de enfermería fueron las variables más expresivas en todas las clases, reforzando el fenómeno investigado: la clasificación de la carga de trabajo de enfermería, que tiene en su esencia la caracterización de la demanda de trabajo requerida de estos profesionales en las actividades diarias de cuidado al paciente ^(^ ^1^ ^)^ . 

 En la práctica clínica de la institución en estudio, estos cuidados prescritos derivan de los diagnósticos realizados por los enfermeros, los cuales se basan en el sistema de lenguaje estandarizado de NANDA-I ^(^ ^8^ ^)^ y se relacionan con el constructo del SCP de Perroca, fundamentado en la identificación de las necesidades de cuidados a los pacientes ^(^ ^3^ ^)^ . Un estudio comparativo entre los DEs y las áreas de cuidado del SCP de Perroca, en el mismo escenario, constató correspondencia entre ambos en al menos una área de cuidado del instrumento, demostrando que los DEs pueden indicar, además de las necesidades de cuidado, el grado de complejidad del cuidado y la dependencia del equipo de enfermería, siendo guías importantes en la gestión del cuidado ^(^ ^25^ ^)^ . 

 Otro aspecto relevante a destacar es la característica de los pacientes del estudio, cuando se compara con la definición de cada clase de cuidado del SCP de Perroca, pues en todas ellas hay variables propias de pacientes dependientes de la enfermería, probablemente debido a la predominancia de personas mayores, lo que refuerza la importancia de los cuidados de enfermería en atención a las necesidades humanas básicas. Este referencial, preconizado en estudios sobre el PE en Brasil, también fundamentó el SCP de Perroca, visto que las necesidades humanas básicas son fenómenos vitales para los seres humanos y, por lo tanto, definidos como entes concretos de la Ciencia de Enfermería ^(^ ^26^ ^-^ ^27^ ^)^ . 

 En la clase de cuidados mínimos, donde, conceptualmente, los pacientes están estables desde el punto de vista clínico y físicamente autosuficientes en cuanto a sus necesidades humanas básicas ^(^ ^3^ ^)^ , el tiempo de internación (período entre la fecha de internación en la unidad hasta la fecha de su evaluación con el SCP de Perroca) fue la variable más importante (4,72%), sugiriendo que, incluso para pacientes que teóricamente requieren menos cuidados, el aumento del tiempo de hospitalización puede generar carga de trabajo para el equipo de enfermería. 

 Llama la atención que la variable de consultoría al servicio social también se destaca, confirmando un perfil de pacientes que, además de cuidados directos de enfermería, poseen problemas que demandan abordajes del equipo multiprofesional, con intervenciones que facilitan la interfaz entre el sistema de cuidados a la salud con el paciente y la familia. Esto demanda una organización que se relaciona directamente con el tiempo de internación del paciente ^(^ ^9^ ^)^ . 

 Un estudio estadounidense, que desarrolló un modelo de AM para mejorar el proceso de alta hospitalaria, identificó como principales barreras potenciales al alta: el paciente que no tiene dieta oral regular, la indisponibilidad de servicios de visita domiciliaria y la falta de apoyo social ^(^ ^28^ ^)^ . Un estudio europeo sobre causas de retraso en el alta hospitalaria refiere, entre los múltiples factores contribuyentes, las dificultades en la transición de cuidados del hospital al hogar o a instituciones de larga estancia ^(^ ^29^ ^)^ . 

 Otras variables surgieron en esta clase de cuidados, pero las características de los pacientes no reflejan la definición de Perroca ^(^ ^3^ ^)^ , porque son diagnósticos y cuidados de enfermería típicos de pacientes dependientes y, aunque fueron clasificados como pertenecientes a la clase de cuidados mínimos, las variables se repiten en las demás clases de cuidados. Ejemplos de esto son el Diagnóstico de Enfermería (DE) déficit de autocuidado ^(^ ^8^ ^)^ , indicando incapacidad para realizar las actividades rutinarias de la vida diaria, tales como alimentación y baño; los cuidados de enfermería relacionados con los dominios fisiológico básico ^(^ ^9^ ^)^ (realizar baño en cama, implementar cuidados con sonda nasoenteral) y fisiológico complejo ^(^ ^9^ ^)^ (implementar protocolo de prevención de tratamiento de heridas); además de la consultoría con fisioterapia. 

 El DE riesgo de caídas ^(^ ^8^ ^)^ , junto con los cuidados del dominio seguridad de los pacientes ^(^ ^9^ ^)^ (mantener las barreras de la cama elevadas, implementar protocolo de prevención de caídas, mantener la cama en posición baja, mantener las ruedas de la cama bloqueadas) representan intervenciones de protección contra la ocurrencia de daños, que en menor o mayor grado forman parte de los cuidados universales para los pacientes hospitalizados. 

 La clase de cuidados intermedios se constituye de pacientes estables bajo el punto de vista clínico y de enfermería, con dependencia parcial de las acciones de enfermería para la atención de las necesidades humanas básicas ^(^ ^3^ ^)^ . En esta clase, la variable que se mostró más importante fue el cuidado de enfermería realizar baño en cama (8,34%), que, asociado con los cuidados realizar higiene después de la evacuación y utilizar pañal desechable, componen cuidados de higiene corporal que demandan esfuerzo físico para movilizar al paciente y cambiar la ropa de cama, pudiendo ocasionar cansancio y lesiones osteomusculares, incluso en los casos en que los profesionales realizan la actividad en pareja ^(^ ^30^ ^)^ . 

 El baño en cama, al tratarse de un cuidado fisiológico básico ^(^ ^9^ ^)^ , forma parte de las actividades diarias del equipo de enfermería y, dependiendo del dimensionamiento de personal y de las condiciones de infraestructura del ambiente, sobrecarga al equipo que realiza esta acción ^(^ ^30^ ^)^ . 

 Entre los demás cuidados que se destacaron están los fisiológicos complejos ^(^ ^9^ ^)^ , relacionados con el DE riesgo de lesión por presión ^(^ ^8^ ^)^ : implementar protocolo de prevención y tratamiento de heridas, proteger la piel y las prominencias óseas, realizar cambio de decúbito e inspeccionar la piel en busca de hiperemia e isquemia ^(^ ^9^ ^)^ . 

 La clase de cuidados semi-intensivos presupone cuidados a pacientes crónicos, estables bajo el punto de vista clínico, pero con total dependencia de las acciones de enfermería en cuanto a la atención de las necesidades humanas básicas ^(^ ^3^ ^)^ . 

En la clasificación realizada por las enfermeras con el SCP de Perroca, 22.106 pacientes fueron clasificados con necesidad de cuidados semi-intensivos, cerca del 50% del primer conjunto de datos.

 Al aplicar el algoritmo RF en el segundo conjunto de datos, la variable principal fue el DE riesgo de caída ^(^ ^8^ ^)^ (3,76%) y varios cuidados relacionados con él ^(^ ^9^ ^)^ . La prevención de caídas es la sexta meta internacional de seguridad, dadas sus altas tasas de incidencia, que corresponden a dos de cada cinco eventos adversos ^(^ ^31^ ^)^ . Las causas de caídas en hospitales son multifactoriales ^(^ ^32^ ^)^ y se relacionan con las demás variables importantes de esta clase de cuidados, tales como la edad media de 62,6, los DE dolor agudo y movilidad física comprometida y los cuidados de enfermería relativos a la herida quirúrgica. Estas variables remiten a los factores de riesgo de la limitación para caminar en el período postoperatorio, los cuales también fueron constatados en diferentes estudios ^(^ ^32^ ^-^ ^36^ ^)^ . 

 La naturaleza de las actividades de enfermería al lado del lecho justifica la preocupación de los profesionales en planificar y ejecutar estrategias de prevención de caídas, aplicando protocolos de seguridad recomendados por órganos gubernamentales nacionales e internacionales, con la asignación de un equipo dedicado a la gestión de riesgos asistenciales, además de estudios sobre el tema ^(^ ^37^ ^)^ . Además, se sabe que el dimensionamiento adecuado de personal y la capacitación de los equipos para la prevención de caídas también influyen en la incidencia de este tipo de evento ^(^ ^35^ ^)^ . 

 La clase de cuidados intensivos comprende cuidados a pacientes graves, con riesgo inminente de vida, sujetos a la inestabilidad de signos vitales, que requieren asistencia de enfermería permanente y especializada ^(^ ^3^ ^)^ . Esta definición de Perroca ^(^ ^3^ ^)^ debe ser relativizada en este estudio, porque los pacientes con inestabilidad clínica grave no permanecen en unidades de internación. Sin embargo, por la opción de presentar las 15 principales variables de cada clase de cuidado, algunas variables no están explícitas en este artículo, tales como las prescripciones y cuidados relacionados con los signos vitales, que aparecen a partir de la vigésima variable, y que son mencionados en la definición de Perroca ^(^ ^3^ ^)^ . 

 El cuidado de enfermería implementar protocolo de prevención y tratamiento de heridas ^(^ ^9^ ^)^ fue la variable más importante en esta clase (7,42%) y, junto con los demás cuidados fisiológicos complejos ^(^ ^9^ ^)^ realizar cambio de decúbito, proteger piel y prominencias óseas e inspeccionar la piel en busca de hiperemia e isquemia, están relacionados con el DE riesgo de lesión por presión ^(^ ^8^ ^)^ . 

 Un estudio global identificó que la ocurrencia de estas lesiones es uno de los eventos adversos más frecuentes en pacientes hospitalizados en todo el mundo, con una prevalencia general del 12,8% ^(^ ^38^ ^)^ y ocurren principalmente en pacientes dependientes, con enfermedades crónicas, hospitalización prolongada, con reducción del sentido de percepción y de la movilidad y con alteraciones nutricionales ^(^ ^39^ ^)^ . Estas características están fundamentadas en diversas variables que se destacaron en la clase de cuidados intensivos: en el DE síndrome de déficit de autocuidado ^(^ ^8^ ^)^ ; en los cuidados de enfermería implementar cuidados con sonda nasoenteral, realizar baño en cama y utilizar pañal desechable; en las prescripciones médicas administrar dieta y medicamentos por sonda nasoenteral; y en la consulta con fisioterapeuta. 

 El riesgo de lesión por presión generalmente es determinado por escalas de predicción aplicadas por enfermeras ^(^ ^40^ ^)^ , pero existen iniciativas de automatización de este proceso, como es el caso de un estudio japonés que desarrolló un modelo predictivo de AM para identificar precozmente el riesgo de lesión por presión, a partir de registros en el expediente electrónico ^(^ ^41^ ^)^ . También es objetivo de este modelo eliminar la carga adicional de trabajo generada por esta actividad ^(^ ^41^ ^)^ , de manera similar a lo propuesto en nuestro estudio de modelo de evaluación de la carga de trabajo de enfermería. 

 Las variables que configuran las cuatro clases del modelo de evaluación de la carga de trabajo de enfermería desarrollado remiten a un contexto asistencial complejo y contemplan las nueve áreas de cuidado del SCP de Perroca ^(^ ^3^ ^)^ , sin embargo, considerando la variable más importante de cada clase, se observan algunas especificidades. 

 La única clase en la que las fragilidades sociales tuvieron destaque fue en los cuidados mínimos y son factores determinantes para el tiempo de hospitalización, guardando relación con las áreas de cuidado soporte emocional y educación a la salud del SCP de Perroca ^(^ ^3^ ^)^ , que requieren una coordinación de cuidados entre equipos asistenciales. 

 En la clase de cuidados intermedios, la variable realizar baño en cama demuestra la fuerte influencia de los cuidados fisiológicos básicos ^(^ ^9^ ^)^ y la relación con el área de cuidado corporal y eliminaciones ^(^ ^3^ ^)^ . 

 Las clases de semi-intensivos e intensivos están dirigidas por los cuidados fisiológicos complejos ^(^ ^9^ ^)^ (prevención/tratamiento de caídas y de lesiones por presión), asociándose a las áreas de locomoción y actividad y de cuidados con piel y mucosas ^(^ ^3^ ^)^ . Ambas requieren intervenciones permanentes de cuidados directos a los pacientes e indirectos, que demandan la organización del ambiente asistencial y colaboración multidisciplinar ^(^ ^3^ ^)^ . 

 Este modelo teórico de evaluación de la carga de trabajo de enfermería retrata la realidad de un hospital universitario de alta complejidad, lo que puede justificar las particularidades de los resultados encontrados. La singularidad de cada escenario asistencial debe considerarse para obtener modelos fiables, sabiendo que, aunque los algoritmos pueden producir predicciones y recomendaciones útiles, el conocimiento y la experiencia humana son esenciales para la toma de decisiones ^(^ ^6^ ^)^ . 

Este estudio tiene limitaciones relacionadas con la capacidad de generalización, porque utiliza datos de pacientes de una única institución. Considerando que los conocimientos sobre aplicaciones de aprendizaje automático diseñadas para la evaluación de la carga de trabajo de enfermería son escasos, se sugiere invertir en la modelización de los datos para mejorar el desempeño del modelo, especialmente en las clases de cuidados semi-intensivos e intensivos, con el fin de que ambos alcancen al menos el 80% de clasificaciones correctas para ser considerados de buen desempeño. Para ello, será importante validar el modelo desarrollado con la recolección de datos electrónicos directamente de la herramienta de evaluación de la carga de trabajo (SCP de Perroca), la cual fue informatizada en el sistema institucional durante la realización de este estudio, proporcionando la cualificación de los datos del patrón oro. Otra sugerencia es probar el modelo con el uso exclusivo de variables con terminologías estandarizadas, fundamentales para investigaciones que involucran ciencia de datos, tales como las taxonomías de los diagnósticos y los cuidados de enfermería (NANDA-I y NIC), lo que podría aumentar la capacidad de buscar patrones en la base de datos analítica y de comprender las clasificaciones realizadas por el modelo.

Se resalta que la replicación del estudio exige estructura tecnológica institucional, disponibilidad de plataformas de ciencias de datos y profesionales con conocimiento en esta área, además de un PE informatizado, utilizando sistema de lenguaje estandarizado. También se destaca que los modelos de clasificación de la carga de trabajo de enfermería, a pesar de ser excelentes orientadores para la gestión asistencial, nunca representarán la totalidad de necesidades de los pacientes por ser innumerables, multidimensionales e interdependientes.

Se cree que el modelo clasificador desarrollado agrega avances al conocimiento científico en enfermería, teniendo en cuenta que es el primer estudio brasileño para evaluar la carga de trabajo de enfermería, usando técnicas de IA, con datos del PEP y utilizando un SCP validado como estándar oro. El estudio es innovador, ya que ese conocimiento es incipiente en Enfermería, y estimula la automatización de procesos, cualificando la gestión asistencial.

## Conclusión

En este estudio se desarrolló un modelo clasificador predictivo de la carga de trabajo de enfermería, utilizando IA. El resultado del modelo demostró que es posible entrenar algoritmos con datos del PEP para predecir la carga de trabajo de enfermería. Las variables tiempo de hospitalización, realizar baño en cama, riesgo de caída e implementar protocolo de prevención y tratamiento de heridas representaron la mayor carga de trabajo en la clasificación final del modelo, dando visibilidad a aspectos relevantes a ser considerados en la planificación de las escalas de actividades del equipo de enfermería y del dimensionamiento de personal en el contexto estudiado. El desarrollo de modelos clasificadores predictivos de la carga de trabajo de enfermería implica la posibilidad de automatizar esta actividad, haciéndola objetiva, precisa y permitiendo que los enfermeros puedan dedicar el tiempo destinado a esta evaluación a otras demandas asistenciales y gerenciales. El modelo predictivo tiene potencial para ser aplicado en diferentes ambientes, siempre que los algoritmos sean entrenados con conjuntos de datos cualificados y que representen la realidad de cada lugar.

# Translation: Carga de trabalho de enfermagem: uso de inteligência artificial para o desenvolvimento de modelo classificador ^*^

**DOI:** 10.1590/1518-8345.7131.4240

**Authors:** Ninon Girardon da Rosa, Tiago Andres Vaz, Amália de Fátima Lucena

**Affiliations:** ^1^ Universidade Federal do Rio Grande do Sul, Escola de Enfermagem, Porto Alegre, RS, Brazil.; ^2^ Hospital de Clínicas de Porto Alegre, Diretoria de Enfermagem, Porto Alegre, RS, Brazil.; ^3^ University Medical Center Utrecht, Data Science and Bioestatistic, Utrecht, Holanda.; ^4^ Hospital de Clínicas de Porto Alegre, Comissão do Processo de Enfermagem, Porto Alegre, RS, Brazil.; ^5^ Bolsista do Conselho Nacional de Desenvolvimento Científico e Tecnológico (CNPq), Brazil.

**Keywords:** Enfermagem, Carga de Trabalho, Informática em Enfermagem, Registros Eletrônicos em Saúde, Inteligência Artificial, Aprendizado de Máquina

## Abstract

**Objetivo:**

descrever o desenvolvimento de um modelo classificador preditivo da carga de trabalho de enfermagem, utilizando inteligência artificial.

**Método:**

estudo observacional retrospectivo, em fontes secundárias de registros eletrônicos de pacientes, com uso de aprendizado de máquina. A amostra por conveniência constituiu-se de 43.871 avaliações realizadas por enfermeiras assistenciais com o Sistema de Classificação de Pacientes de Perroca, as quais serviram como padrão ouro, e os dados clínicos do prontuário eletrônico de 11.774 pacientes, que constituíram as variáveis. Para a organização dos dados e a realização das análises, utilizou-se a plataforma de ciência de dados Dataiku ^®^ . A análise dos dados ocorreu de forma exploratória, descritiva e preditiva. Estudo aprovado pelo Comitê de Ética e Pesquisa da instituição campo do estudo.

**Resultados:**

o uso de inteligência artificial possibilitou o desenvolvimento do modelo classificador de avaliação da carga de trabalho de enfermagem, identificando as variáveis que mais contribuíram para a sua predição. O algoritmo classificou corretamente 72% das variáveis e a área sob a curva *Receiver Operating Characteristic* foi de 82%.

**Conclusão:**

houve o desenvolvimento de um modelo preditivo, demonstrando que é possível treinar algoritmos com dados do prontuário eletrônico do paciente para predizer a carga de trabalho de enfermagem e que as ferramentas da inteligência artificial podem ser efetivas para a automatização desta atividade.

## Destaques

**(1)** Desenvolvimento de modelo classificador preditivo da carga de trabalho de enfermagem.

**(2)** Identificação das principais variáveis que geram carga de trabalho de enfermagem.

**(3)** Possibilidade de automatização da avaliação da carga de trabalho de enfermagem.

**(4)** Qualificação da gestão assistencial.

**(5)** Contribuição nos estudos de dimensionamento de pessoal.

## Introdução

 A carga de trabalho em enfermagem caracteriza-se pela demanda de trabalho requerida dos profissionais de enfermagem nas atividades de cuidado ao paciente ^(^ ^1^ ^)^ . Existem sistemas de classificação que categorizam os pacientes de acordo com a quantidade, complexidade e tempo despendido para o cuidado. Diversos sistemas de classificação são utilizados no mundo, não havendo base científica para que um sistema tenha preferência sobre o outro, mas todos visam estimar a carga de trabalho, proporcionando parâmetros para o planejamento dos recursos assistenciais necessários ^(^ ^2^ ^)^ . 

 No Brasil, o Sistema de Classificação de Pacientes (SCP) de Perroca é utilizado para pacientes adultos hospitalizados em unidades de internação. São consideradas nove áreas de cuidado na avaliação: planejamento e coordenação do processo de cuidar; investigação e monitoramento; cuidado corporal e eliminações; cuidados com pele e mucosas; nutrição e hidratação; locomoção e atividade; terapêutica; suporte emocional e educação à saúde. Cada indicador possui uma gradação de 1 a 4, indicando a complexidade crescente do cuidado. Esses valores somados levam à classificação em uma das quatro categorias de cuidados: mínimos, intermediários, semi-intensivos e intensivos, os quais determinam o grau de dependência do paciente em relação aos cuidados de enfermagem ^(^ ^3^ ^)^ . 

 A avaliação contínua da carga de trabalho traz implicações positivas ao gerenciamento do processo de trabalho dos enfermeiros, embora tenha um grau de subjetividade e exija tempo dedicado para a aplicação dos instrumentos, em meio a tantas atribuições assistenciais e gerenciais desenvolvidas ^(^ ^4^ ^)^ . Tal contexto demonstra a importância do aprimoramento desse processo, pois a avaliação da carga de trabalho fornece parâmetros para o dimensionamento de pessoal das instituições brasileiras de saúde ^(^ ^5^ ^)^ . Um quadro de pessoal equilibrado proporciona a realização de cuidados essenciais aos pacientes, a prevenção de eventos adversos, além de segurança aos profissionais e satisfação no trabalho ^(^ ^2^ ^)^ . 

 Nos hospitais, o investimento crescente no Prontuário Eletrônico do Paciente (PEP) possibilita a sistematização dos dados oriundos dos registros dos atendimentos dos profissionais e a utilização de técnicas inovadoras de processamento ágil de grandes volumes de dados clínicos e análises preditivas, além do desenvolvimento de ferramentas que automatizam atividades ^(^ ^6^ ^)^ . 

 Assim, a aproximação dos enfermeiros com a ciência de dados amplia as possibilidades de desenvolver soluções inovadoras, com informações úteis, extraídas dos bancos de dados ^(^ ^7^ ^)^ , principalmente nas instituições em que há um ambiente favorável à informatização de todas as etapas do Processo de Enfermagem (PE), o que promove a sistematização de dados e uso de linguagem padronizada, tais como os Diagnósticos de Enfermagem (DEs) da NANDA Internacional (NANDA-I) ^(^ ^8^ ^)^ e as intervenções e atividades da Classificação das Intervenções de Enfermagem (NIC) ^(^ ^9^ ^)^ . 

 Os bancos de dados analíticos introduzem os conceitos relacionados a volumes de dados, velocidade, variedade, veracidade e valor, que caracterizam a definição de *Big Data* , demonstrando identidade com o contexto dinâmico dos hospitais, que estão se beneficiando do desenvolvimento de sistemas inteligentes ^(^ ^7^ ^)^ . 

 A Inteligência Artificial (IA) pode desenvolver atividades com algum grau de autonomia para alcançar objetivos específicos, por isso o seu uso na enfermagem pode oferecer assertividade de dados e redução no tempo de trabalho ^(^ ^10^ ^)^ . 

 As técnicas de IA, como o aprendizado de máquina, auxiliam nas respostas dos problemas de enfermagem, assim, os enfermeiros engajados nessa discussão mundial elevam a qualidade da prática e da gestão assistencial e têm muito a contribuir no desenvolvimento de sistemas informatizados e desenvolvimento de modelos preditivos ^(^ ^10^ ^)^ . 

 Esses avanços têm sido impulsionados por estudos multidisciplinares com profissionais da saúde e cientistas de dados, que buscam desenvolver modelos que atendam à complexidade do ambiente hospitalar ^(^ ^7^ ^,^ ^10^ ^-^ ^11^ ^)^ . 

 Embora os hospitais brasileiros necessitem enfrentar desafios relacionados à infraestrutura tecnológica, sistematização de dados e interoperabilidade entre sistemas para a utilização das ferramentas de IA, a liderança dos enfermeiros tem um papel fundamental na busca de inovações e no desenvolvimento de competências, para que os profissionais da área possam estar preparados para um mundo cada vez mais informatizado ^(^ ^11^ ^)^ . 

 A aplicação da IA em ambientes de cuidado de enfermagem é recente e tem sido explorada principalmente nos hospitais, em processamento de imagens e sinais, em classificação de atividades de enfermagem, na comunicação de cuidados e na detecção de quedas. A identificação de outras áreas de aplicação desta técnica pode gerar importantes contribuições para o ensino, para a pesquisa e para a prática de enfermagem ^(^ ^12^ ^)^ . 

 No que tange à avaliação de carga de trabalho de enfermagem, em revisão da literatura, não foram encontrados estudos brasileiros que aplicam IA para a realização dessa mensuração. Escassas publicações internacionais abordam a utilização de instrumentos automatizados ^(^ ^13^ ^-^ ^15^ ^)^ , no entanto, não há menção sobre o uso de dados gerados pelo PE, que é uma metodologia de referência à organização da assistência e dos registros de enfermagem ^(^ ^16^ ^)^ . 

 A Associação Norte Americana de Enfermagem (ANA) estimula o desenvolvimento de sistemas de avaliação de carga de trabalho de enfermagem automatizados, destacando a importância do embasamento científico e da capacidade de integração com as plataformas institucionais. A ANA ressalta que os instrumentos devem ser simples e eficientes, para traduzir a realidade de cada local e não gerar carga de trabalho adicional aos enfermeiros, mas precisam ser validados e seus resultados monitorados a fim de oferecerem predições corretas sobre as necessidades das equipes e dos pacientes ^(^ ^17^ ^)^ . A operacionalização das recomendações depende do desenvolvimento de pesquisas conjuntas entre profissionais da saúde e cientistas de dados, de forma a superar a lacuna de conhecimentos e as eventuais fragilidades no uso de dados secundários e IA ^(^ ^17^ ^)^ . 

Baseando-se nessas ideias e na carência de pesquisas sobre o tema, este artigo tem como objetivo descrever o desenvolvimento de um modelo classificador preditivo da carga de trabalho de enfermagem, utilizando IA.

## Método

### Tipo de estudo

 Trata-se de um estudo observacional retrospectivo, em fontes secundárias de registros eletrônicos de pacientes, com elaboração de modelo classificador preditivo com técnicas supervisionadas de Aprendizado de Máquina (AM) ^(^ ^18^ ^)^ . 

### Cenário do estudo

O estudo ocorreu de 2021 a 2023, em um hospital público universitário de grande porte, com atendimento a pacientes de alta complexidade. A instituição é acreditada internacionalmente em padrões de qualidade e segurança e tem como pilares a assistência, o ensino, a pesquisa de excelência e a inovação. O hospital desenvolveu um sistema informatizado próprio na década de 80, o qual vem sendo aprimorado desde então. O PE e os instrumentos de avaliação de carga de trabalho já estão incorporados, mas não possuem interoperabilidade entre si.

### Logística dos dados

 Os pesquisadores utilizaram as etapas do processo de *Knowledge Discovery in Databases* (KDD) ^(^ ^18^ ^)^ como roteiro na logística dos dados: seleção, pré-processamento, transformação e mineração dos dados e interpretação de resultados. O KDD ^(^ ^19^ ^)^ é um processo para identificar padrões consistentes em grande quantidade de dados e descobrir informações relevantes para apoiar decisões estratégicas. 

### Estruturação do banco de dados analítico

 A estruturação do banco de dados analítico envolve seleção, pré-processamento e transformação dos dados ^(^ ^19^ ^)^ . 

 Na etapa de seleção ocorreu a definição das informações do estudo, organizadas em dois conjuntos, seguida da coleta de dados e da sua importação para a plataforma de ciência de dados Dataiku ^®^ , associada ao *software* de processamento de *Big Data* PostgreSQL ^®^ , o qual permitiu o gerenciamento do banco de dados e o relacionamento de dados complexos entre si. 

 O primeiro conjunto foi organizado a partir de 107.507 avaliações de pacientes realizadas por enfermeiras assistenciais de doze unidades de internação clínico-cirúrgicas, no período de 2015 a 2019, utilizando o SCP de Perroca ^(^ ^3^ ^)^ . Esses dados correspondem aos escores dos pacientes e às respectivas classificações nas categorias de cuidados mínimos, intermediários, semi-intensivos e intensivos. As planilhas continham o número do leito do paciente e a data da avaliação. Os dados foram coletados pelos pesquisadores diretamente das planilhas informatizadas do sistema da instituição e foram organizados em planilha única para serem usados como padrão ouro. 

 O segundo conjunto foi organizado a partir de dados extraídos do PEP de pacientes internados nas mesmas unidades, no mesmo período do primeiro conjunto de dados. A solicitação de consulta aos prontuários ( *query* ) foi planejada em reuniões com a analista de Tecnologia da Informação (TI) que realiza essa atividade na instituição, considerando as variáveis em estudo. Assim, em cada *query* foi solicitada uma variável representativa da carga de trabalho de enfermagem, com base nas nove áreas de cuidado do SCP de Perroca ^(^ ^3^ ^)^ , quais sejam: cuidados de enfermagem; diagnósticos de enfermagem; registros dos sinais vitais; prescrições de dieta; prescrição de medicamentos; prescrição de soluções; prescrição de hemoterapia; prescrição de oxigenoterapia; solicitações de exames coletados pela enfermagem; solicitações de consultorias por especialistas e existência de condutas educativas a pacientes e familiares. 

 Em todas as *queries* também foram solicitadas as variáveis idade, sexo, escolaridade, data da internação e os identificadores número do leito e do prontuário. Assim, o estudo contemplou 15 diferentes variáveis que se ampliaram considerando suas estratificações, como por exemplo: os cuidados de enfermagem totalizaram 846 e os diagnósticos de enfermagem totalizaram 189 variáveis. Os dados disponibilizados em tabelas foram referentes a 58.888 pacientes internados. Dentre eles, foram selecionados aqueles que estavam cadastrados no mesmo leito e na mesma data de internação dos pacientes do primeiro conjunto de dados, o que resultou em 11.774 pacientes. 

Não foram incluídos, no modelo, os dados anteriores a 2015, por não haver registros sistematizados de avaliação dos pacientes com o SCP de Perroca e dados posteriores a 2019, porque os processos assistenciais foram alterados durante a pandemia causada pelo coronavírus.

Na etapa de pré-processamento, os pesquisadores realizaram revisão e limpeza para garantir a qualidade dos dados.

 Para o primeiro conjunto de dados, os critérios de exclusão adotados foram: pacientes diferentes que tinham avaliações com SCP de Perroca ^(^ ^3^ ^)^ no mesmo dia e estavam cadastrados no mesmo leito, considerando a possibilidade de dúvida se a avaliação registrada era do paciente que saiu ou do paciente que entrou no leito; pacientes com duas avaliações diferentes no mesmo dia e que estavam cadastrados no mesmo leito também foram excluídos, devido à possibilidade de erro ao registrar o leito do paciente ou pela possibilidade de ser um segundo registro relativo ao mesmo paciente para corrigir um equívoco de avaliação, ao invés da correção ter sido efetuada no primeiro registro; pacientes com menos de 48 horas de internação no momento da avaliação, pois inferiu-se que esses pacientes teriam menos chances de se exporem às variáveis estabelecidas para a análise. Após a aplicação desses critérios, no conjunto de 107.507 avaliações, obteve-se 43.871 avaliações para o estudo. 

Em relação ao segundo conjunto de dados, a etapa de pré-processamento exigiu bastante tempo e dedicação dos pesquisadores na revisão, no entanto a reorganização de tabelas não implicou em alteração do número de pacientes, corroborando os 11.774 pacientes.

 Os dados dos dois conjuntos foram importados para a plataforma de ciência de dados Dataiku ^®^ à medida que foram sendo disponibilizados. 

 Na etapa de transformação, os dados brutos foram processados para terem uniformidade e serem minerados com técnicas de AM, em busca de padrões e relações no grande conjunto de dados ( *Big Data* ). 

### Mineração dos dados

 Mineração de dados é o processo de analisar grandes conjuntos para identificar padrões e extrair informações que geram conhecimentos e apoiam decisões ^(^ ^19^ ^)^ . Na mineração de dados, são usados algoritmos de AM, que definem a sequência dos procedimentos por meio de instruções matemáticas automatizadas ^(^ ^18^ ^)^ . Este estudo foi desenvolvido na linguagem *Python* , com o módulo de algoritmos de classificação do pacote *Scikit-learn* ^(^ ^20^ ^)^ . O algoritmo aplicado foi o *Random Forest* (RF), caracterizado por uma combinação de preditores que se desenvolvem em forma de árvores de decisão e se distribuem nas classes conforme o grau de pureza e de homogeneidade ^(^ ^21^ ^)^ . 

 No desenvolvimento do modelo preditivo foi utilizada a técnica de validação cruzada *k-fold* (k=5), com divisão do banco de dados analítico em k partições para o modelo ser treinado em k-1 partições e validado na partição restante. Esse procedimento foi repetido, alternando a partição dos dados de treino ^(^ ^22^ ^)^ , buscando acertar a classe de cuidados dos pacientes (mínimo, intermediário, intensivo, semi-intensivo). 

 O processo incluiu estratégias para garantir a qualidade das etapas: aprimoramentos nos parâmetros do algoritmo RF, os quais atingiram uma composição final de 1000 árvores, com seis camadas de profundidade ^(^ ^21^ ^)^ , e checagens para evitar *overfitting* (ajuste excessivo que ocorre quando o modelo de aprendizado de máquina fornece previsões precisas apenas para dados de treinamento, como se o modelo não fosse capaz de generalizar para os outros dados não vistos) e vazamento de dados (compartilhamento de dados entre as fases de treinamento e validação). Esses ajustes elevaram a acurácia até o ponto de saturação de desempenho das métricas do modelo ^(^ ^22^ ^)^ . 

 As métricas avaliadas resultaram da matriz de confusão (acurácia, sensibilidade, F1 *score* e precisão) e do desempenho da curva ROC ( *Receiver Operating Characteristic* ) e da AUC-ROC ( *Area Under The ROC Curve* ) ^(^ ^22^ ^)^ . 

 A importância de cada variável para a classificação final da carga de trabalho de enfermagem foi conhecida na fase de predição do modelo ^(^ ^23^ ^)^ . 

### Análise dos dados

 Para a análise, utilizou-se a plataforma de ciência de dados Dataiku ^®^ e o *software* de processamento de *Big Data* PostgreSQL ^®^ . A análise exploratória foi realizada em todas as etapas da pesquisa para a compreensão dos dados e das relações entre as variáveis ^(^ ^19^ ^)^ . A análise descritiva foi utilizada para detalhar as características dos conjuntos de dados e os resultados das variáveis foram apresentados em frequência relativa. A análise preditiva ocorreu na fase de interpretação dos resultados, possibilitando explorar as relações entre variáveis de eventos anteriores e variáveis previstas no modelo desenvolvido, além de conhecer o desempenho das métricas correspondentes ^(^ ^18^ ^)^ . 

### Aspectos éticos

O estudo foi aprovado pelo Comitê de Ética e Pesquisa institucional (2021-0521) e cadastrado na Plataforma Brasil (55207921.50000.5327). As recomendações do Termo de Compromisso de Utilização de Dados foram adotadas pelos pesquisadores e houve dispensa de uso do Termo de Consentimento Livre e Esclarecido.

## Resultados

### Caracterização das avaliações dos pacientes realizadas por enfermeiras com o SCP de Perroca, referente ao primeiro conjunto de dados

As avaliações realizadas pelas enfermeiras compreenderam 43.871 registros. A maioria dos pacientes está na classe de cuidados semi-intensivos (50,61%), seguida da classe de cuidados intermediários (31,25%). A idade média é de 61 anos [Desvio-Padrão (DP)±16,4], 50,08% são do sexo masculino e 52,77% possuem o ensino fundamental.

### Caracterização dos pacientes dos quais foram extraídos dados dos prontuários eletrônicos para compor as variáveis do segundo conjunto de dados

Os pacientes internados nas unidades clínico-cirúrgicas caracterizaram-se por 11.774 pessoas, com idade média de 61,6 anos (DP ±16,7), 50,95%, do sexo masculino e 54,39% tem ensino fundamental.

### Importância das variáveis no modelo preditor de avaliação da carga de trabalho de enfermagem

 O algoritmo RF foi treinado para classificar as variáveis nas quatro classes de cuidados do SCP de Perroca ^(^ ^3^ ^)^ . O número total de variáveis que compõem cada classe é diferente: 574 para mínimos, 701 para intermediários, 731 para semi-intensivos e 692 para intensivos. Neste artigo, apresenta-se as 15 variáveis mais importantes para cada classe, segundo o grau de importância no modelo, conforme as Figuras 1 , 2 , 3 e 4 . 

**Figura 1:** - Descrição da importância das variáveis na classe de cuidados mínimos do modelo preditor de avaliação da carga de trabalho de enfermagem (n = 574). Porto Alegre, RS, Brasil, 2023

*SNE = Sonda Nasoenteral

**Figura 2:** - Descrição da importância das variáveis na classe de cuidados intermediários do modelo preditor de avaliação da carga de trabalho de enfermagem (n = 701). Porto Alegre, RS, Brasil, 2023

*SNE = Sonda Nasoenteral

**Figura 3:** - Descrição da importância das variáveis na classe de cuidados semi-intensivos do modelo preditor de avaliação da carga de trabalho de enfermagem (n = 731). Porto Alegre, RS, Brasil, 2023

**Figura 4:** - Descrição da importância das variáveis na classe de cuidados intensivos no modelo preditor de avaliação da carga de trabalho de enfermagem (n = 692). Porto Alegre, RS, Brasil, 2023

^*^ SNE = Sonda Nasoenteral

### Métricas de avaliação do desempenho do modelo classificador

 A partir das classificações das variáveis, o modelo buscou identificar as previsões positivas corretas. Os resultados das métricas estão demonstrados na Tabela 1 . 

 Todas as métricas tiveram excelentes desempenhos na classe de cuidados intermediários (0,962), seguida da classe de cuidados mínimos (0,917). Isso significa que o algoritmo fez uma boa distinção dos cuidados requeridos para os pacientes dessas duas classes. As classes de cuidados semi-intensivos (0,759) e intensivos (0,791) tiveram menores resultados. Os modelos com desempenho entre 0,8 < 0,9 são considerados bons e aqueles com desempenho ≥ 0,9 são excelentes ^(^ ^21^ ^)^ . 

**Tabela 1:** - Métricas de avaliação do desempenho do modelo classificador preditivo da carga de trabalho de enfermagem, segundo a classificação em cuidados mínimos, intermediários, semi-intensivos e intensivos do SCP ^*^ de Perroca. Porto Alegre, RS, Brasil, 2023

**SCP** ^*^ **Perroca**	**Acurácia**	**Sensibilidade**	**Precisão**	**F1** **Score** ^†^	**AUC- ROC** ^‡^
Mínimos	0,990	0,715	0,849	0,776	0,917
Intermediários	0,952	0,929	0,917	0,923	0,962
Semi-intensivos	0,691	0,965	0,625	0,759	0,759
Intensivos	0,824	0,463	0,459	0,461	0,791
Modelo final	0,72	0,66	0,67	0,64	0,82

^*^ SCP Perroca = Sistema de Classificação de Pacientes de Perroca;

^†^ F1 Score = *Function 1 Score* ;

^‡^ AUC-ROC = *Area Under The Receiver Operating Characteristic Curve*

 Os valores do modelo final têm base na abordagem *Multiclass Area Under the Curve* , que classificou as quatro classes ao mesmo tempo e apresentou DP de ± 0,036 ^(^ ^21^ ^-^ ^24^ ^)^ . 

 De forma global, o algoritmo RF classificou corretamente 72% das variáveis do modelo e a AUC-ROC indicou que o modelo teve um desempenho de 82% na classificação geral dos pacientes nas diferentes categorias de cuidados ^(^ ^21^ ^)^ . 

## Discussão

 Observa-se que a maioria dos pacientes avaliados com o SCP de Perroca ^(^ ^3^ ^)^ são pessoas idosas, com ensino fundamental e fazem parte da classe de cuidados semi-intensivos. Identifica-se também que, na classificação dos registros do PEP, o algoritmo RF selecionou variáveis semelhantes para compor as classes de cuidados, no entanto atribuiu a elas diferentes graus de importância. Os cuidados de enfermagem foram as variáveis mais expressivas em todas as classes, reforçando o fenômeno pesquisado: a classificação da carga de trabalho de enfermagem, cuja essência é a caracterização da demanda de trabalho requerida desses profissionais nas atividades diárias de cuidado ao paciente ^(^ ^1^ ^)^ . 

 Na prática clínica da instituição em estudo, os cuidados prescritos decorrem dos diagnósticos realizados pelos enfermeiros, os quais se baseiam no sistema de linguagem padronizada da NANDA-I ^(^ ^8^ ^)^ e se relacionam com o construto do SCP de Perroca, fundamentado na identificação das necessidades de cuidados aos pacientes ^(^ ^3^ ^)^ . Um estudo comparativo entre os DEs e as áreas de cuidado do SCP de Perroca, no mesmo cenário, constatou correspondência entre ambos em pelo menos uma área de cuidado do instrumento, demonstrando que os DEs podem indicar, além das necessidades de cuidado, o grau da complexidade demandado e a dependência da equipe de enfermagem, sendo guias importantes na gestão do cuidado ^(^ ^25^ ^)^ . 

 Outro aspecto relevante a ser ressaltado é a característica dos pacientes do estudo, quando comparada à definição de cada classe de cuidado do SCP de Perroca, pois, em todas elas há variáveis próprias de pacientes dependentes da enfermagem, provavelmente devido à predominância de pessoas idosas, o que reforça a importância dos cuidados de enfermagem em atenção às necessidades humanas básicas. Este referencial, preconizado em estudos sobre PE no Brasil, também alicerçou o SCP de Perroca, visto que as necessidades humanas básicas são fenômenos vitais aos seres humanos e, portanto, definidas como entes concretos da Ciência de Enfermagem ^(^ ^26^ ^-^ ^27^ ^)^ . 

 Na classe de cuidados mínimos, em que, conceitualmente, os pacientes estão estáveis do ponto de vista clínico e fisicamente autossuficientes quanto às suas necessidades humanas básicas ^(^ ^3^ ^)^ , o tempo de internação (período entre a data da internação na unidade até a data da sua avaliação com o SCP de Perroca) foi a variável mais importante (4,72%), sugerindo que, mesmo para pacientes que teoricamente exigem menos cuidados, o aumento do tempo de hospitalização pode gerar carga de trabalho para a equipe de enfermagem. 

 Chama atenção que a variável consultoria ao serviço social também se destaca, confirmando um perfil de pacientes que, para além de cuidados diretos de enfermagem, possuem problemas que demandam abordagens da equipe multiprofissional, com intervenções que facilitem a interface entre o sistema de cuidados à saúde com o paciente e a família. Isso requer uma organização que se relaciona diretamente com o tempo de internação do paciente ^(^ ^9^ ^)^ . 

 Um outro estudo norte-americano, que desenvolveu um modelo de AM para melhorar o processo de alta hospitalar, identificou como principais barreiras potenciais à alta: o paciente que não tem dieta oral regular, a indisponibilidade de serviços de visita domiciliar e a falta de apoio social ^(^ ^28^ ^)^ . Além disso, um estudo europeu sobre causas de atraso na alta hospitalar refere, entre os múltiplos fatores contribuintes, as dificuldades na transição de cuidados do hospital para o domicílio ou para instituições de longa permanência ^(^ ^29^ ^)^ . 

 Outras variáveis surgiram nessa classe de cuidados, mas as características dos pacientes não traduzem a definição de Perroca ^(^ ^3^ ^)^ porque são diagnósticos e cuidados de enfermagem típicos de pacientes dependentes e, embora eles tenham sido classificados como pertencentes a classe de cuidados mínimos, as variáveis se repetem nas demais classes de cuidados. Exemplos disso são o Diagnóstico de Enfermagem (DE) déficit para o autocuidado ^(^ ^8^ ^)^ , indicando incapacidade para realizar as atividades de rotina da vida diária, tais como alimentação e banho; os cuidados de enfermagem relacionados aos domínios fisiológico básico ^(^ ^9^ ^)^ (realizar banho de leito, implementar cuidados com sonda nasoenteral) e fisiológico complexo ^(^ ^9^ ^)^ (implementar protocolo de prevenção de tratamento de feridas); além da consultoria com a fisioterapia. 

 O DE risco de quedas ^(^ ^8^ ^)^ , junto com os cuidados do domínio segurança dos pacientes ^(^ ^9^ ^)^ (manter grades do leito elevadas, implementar protocolo de prevenção de quedas, manter cama em posição baixa, manter rodas da cama travadas), representam intervenções de proteção contra a ocorrência de danos, que em menor ou maior grau fazem parte de cuidados universais aos pacientes hospitalizados. 

 A classe de cuidados intermediários constitui-se de pacientes estáveis sob o ponto de vista clínico e de enfermagem, com parcial dependência das ações de enfermagem para o atendimento das necessidades humanas básicas ^(^ ^3^ ^)^ . Nessa classe, a variável que se mostrou mais importante foi o cuidado de enfermagem ao realizar banho de leito (8,34%), que, associado com os cuidados realizar higiene após evacuação e utilizar fralda descartável, compõem cuidados de higiene corporal que demandam esforço físico para mobilizar o paciente e trocar a roupa de cama, podendo ocasionar cansaço e lesões osteomusculares, mesmo nos casos em que os profissionais realizam a atividade em dupla ^(^ ^30^ ^)^ . 

 O banho de leito, por tratar-se de um cuidado fisiológico básico ^(^ ^9^ ^)^ , faz parte das atividades diárias da equipe de enfermagem e, dependendo do dimensionamento de pessoal e das condições de infraestrutura do ambiente, sobrecarrega a equipe que realiza a ação ^(^ ^30^ ^)^ . 

 Dentre os demais cuidados que se destacaram estão os fisiológicos complexos ^(^ ^9^ ^)^ , relacionados com o DE risco de lesão por pressão ^(^ ^8^ ^)^ : implementar protocolo de prevenção e tratamento de feridas, proteger pele e proeminências ósseas, realizar mudança de decúbito e inspecionar pele em busca de hiperemia e isquemia ^(^ ^9^ ^)^ . 

 A classe dos cuidados semi-intensivos pressupõe cuidados a pacientes crônicos, estáveis sob o ponto de vista clínico, porém, com total dependência das ações de enfermagem quanto ao atendimento das necessidades humanas básicas ^(^ ^3^ ^)^ . 

Na classificação realizada pelas enfermeiras com o SCP de Perroca, 22.106 pacientes foram classificados com necessidade de cuidados semi-intensivos, cerca de 50% do primeiro conjunto de dados.

 Ao aplicar o algoritmo RF no segundo conjunto de dados, a variável principal foi o DE risco de queda ^(^ ^8^ ^)^ (3,76%) e vários cuidados a ele relacionados ^(^ ^9^ ^)^ . A prevenção de quedas é a sexta meta internacional de segurança, tendo em vista as suas altas taxas de incidência, que correspondem a dois em cada cinco eventos adversos ^(^ ^31^ ^)^ . As causas de quedas em hospitais são multifatoriais ^(^ ^32^ ^)^ e relacionam-se com as demais variáveis importantes dessa classe de cuidados, tais como idade média de 62,6 anos, os DEs dor aguda e mobilidade física prejudicada e os cuidados de enfermagem relativos à ferida operatória. Estas variáveis remetem aos fatores de risco da limitação para caminhar no período pós-operatório, os quais também foram constatados em diferentes estudos ^(^ ^32^ ^-^ ^36^ ^)^ . 

 A natureza das atividades de enfermagem à beira leito justifica a preocupação dos profissionais em planejar e executar estratégias de prevenção de quedas, aplicando protocolos de segurança recomendados por órgãos governamentais nacionais e internacionais, com alocação de equipe dedicada à gestão de riscos assistenciais, além de estudos sobre o tema ^(^ ^37^ ^)^ . Ademais, sabe-se que o dimensionamento adequado de pessoal e a capacitação das equipes para a prevenção de quedas também influenciam na incidência desse tipo de evento ^(^ ^35^ ^)^ . 

 A classe de cuidados intensivos compreende cuidados a pacientes graves, com risco iminente de vida, sujeitos à instabilidade de sinais vitais, que requeiram assistência de enfermagem permanente e especializada ^(^ ^3^ ^)^ . Essa definição de Perroca ^(^ ^3^ ^)^ deve ser relativizada neste estudo, porque pacientes com instabilidade clínica grave não ficam em unidades de internação. No entanto, pela opção de apresentar as 15 principais variáveis de cada classe de cuidado, algumas variáveis não estão explicitadas neste artigo, tais como as prescrições e cuidados relacionados aos sinais vitais, que aparecem a partir da vigésima variável, e que são mencionados na definição de Perroca ^(^ ^3^ ^)^ . 

 O cuidado de enfermagem implementar protocolo de prevenção e tratamento de feridas ^(^ ^9^ ^)^ foi a variável mais importante na classe (7,42%) e, junto com os demais cuidados fisiológicos complexos ^(^ ^9^ ^)^ , como realizar mudança de decúbito, proteger pele e proeminências ósseas e inspecionar pele em busca de hiperemia e isquemia, estão relacionados ao DE risco de lesão por pressão ^(^ ^8^ ^)^ . 

 Um estudo global identificou que a ocorrência de lesões é um dos eventos adversos mais frequentes em pacientes hospitalizados em todo o mundo, com prevalência geral de 12,8% ^(^ ^38^ ^)^ , ocorrendo principalmente em pacientes dependentes, com doenças crônicas, internação prolongada, com redução do senso de percepção e da mobilidade e com alterações nutricionais ^(^ ^39^ ^)^ . Tais características estão fundamentadas em diversas variáveis que se destacaram na classe de cuidados intensivos: no DE síndrome de déficit autocuidado ^(^ ^8^ ^)^ ; nos cuidados de enfermagem implementar cuidados com sonda nasoenteral, realizar banho de leito e utilizar fralda descartável; assim como nas prescrições médicas administrar dieta e medicamentos por sonda nasoenteral e na consultoria com fisioterapeuta. 

 O risco de lesão por pressão geralmente é determinado por escalas de predição aplicadas por enfermeiras ^(^ ^40^ ^)^ , mas existem iniciativas de automatização desse processo, como é o caso de um estudo japonês que desenvolveu um modelo preditivo de AM para identificar precocemente o risco de lesão por pressão, a partir de registros no prontuário eletrônico ^(^ ^41^ ^)^ . Também é objetivo desse modelo a retirada da carga adicional de trabalho gerada pela atividade ^(^ ^41^ ^)^ , de modo semelhante ao proposto em nosso estudo de modelo de avaliação da carga de trabalho de enfermagem. 

 As variáveis que configuram as quatro classes do modelo de avaliação da carga de trabalho de enfermagem desenvolvido remetem a um contexto assistencial complexo e contemplam as nove áreas de cuidado do SCP de Perroca ^(^ ^3^ ^)^ , no entanto, considerando a variável mais importante de cada classe, observam-se algumas especificidades. 

 A única classe em que as fragilidades sociais tiveram destaque foi a dos cuidados mínimos e elas são fatores determinantes para o tempo de internação, guardando relação com as áreas de cuidado suporte emocional e educação à saúde do SCP de Perroca ^(^ ^3^ ^)^ , que demandam uma coordenação de cuidados entre equipes assistenciais. 

 Na classe dos cuidados intermediários, a variável realizar banho de leito demonstra a forte influência dos cuidados fisiológicos básicos ^(^ ^9^ ^)^ , bem como a relação com a área cuidado corporal e eliminações ^(^ ^3^ ^)^ . 

 As classes dos semi-intensivos e intensivos são direcionadas pelos cuidados fisiológicos complexos ^(^ ^9^ ^)^ (prevenção/tratamento de quedas e de lesões por pressão), associando-se às áreas de locomoção e à atividade e de cuidados com pele e mucosas ^(^ ^3^ ^)^ . Ambas exigem intervenções permanentes de cuidados diretos aos pacientes e também indiretos, os quais demandam a organização do ambiente assistencial e colaboração multidisciplinar ^(^ ^3^ ^)^ . 

 Este modelo teórico de avaliação de carga de trabalho de enfermagem retrata a realidade de um hospital universitário de alta complexidade, o que pode justificar as particularidades dos resultados encontrados. A singularidade de cada cenário assistencial deve ser considerada para se obter modelos confiáveis, sabendo-se que, embora os algoritmos possam produzir previsões e recomendações úteis, o conhecimento e a experiência humana são essenciais para a tomada de decisão ^(^ ^6^ ^)^ . 

Este estudo tem limitações relacionadas à capacidade de generalização, porque utiliza dados de pacientes de uma única instituição. Considerando que são escassos os conhecimentos sobre aplicativos de aprendizado de máquina projetados para avaliação de carga de trabalho de enfermagem, sugere-se investir na modelagem dos dados para melhorar o desempenho do modelo, especialmente nas classes de cuidados semi-intensivos e intensivos, no intuito de ambos alcançarem o mínimo de 80% de classificações corretas para serem considerados com bom desempenho. Para isso, será importante validar o modelo desenvolvido com a coleta de dados eletrônicos diretamente da ferramenta de avaliação da carga de trabalho (SCP de Perroca), a qual foi informatizada no sistema institucional durante a realização deste estudo, proporcionando a qualificação dos dados do padrão ouro. Outra sugestão é testar o modelo com o uso exclusivo de variáveis com terminologias padronizadas, fundamentais para pesquisas que envolvem ciência de dados, tais como as taxonomias dos diagnósticos e dos cuidados de enfermagem (NANDA-I e NIC), o que poderá aumentar a capacidade de buscar padrões no banco de dados analítico e de compreender as classificações realizadas pelo modelo.

Destaca-se que a replicação do estudo exige estrutura tecnológica institucional, disponibilidade de plataformas de ciências de dados e profissionais com conhecimento na área, além de PE informatizado, utilizando sistema de linguagem padronizada. Ressalta-se, ainda, que os modelos de classificação de carga de trabalho de enfermagem, apesar de serem excelentes norteadores para a gestão assistencial, nunca representarão a totalidade de necessidades dos pacientes por serem inúmeras, multidimensionais e interdependentes.

Acredita-se que o modelo classificador desenvolvido agrega avanços ao conhecimento científico na enfermagem, tendo em vista que é o primeiro estudo brasileiro para avaliar carga de trabalho de enfermagem usando técnicas de IA, com dados do PEP e utilizando um SCP validado como padrão ouro. O estudo é inovador, visto que tal conhecimento é incipiente na Enfermagem, e estimula a automatização de processos, qualificando a gestão assistencial.

## Conclusão

Neste estudo, foi desenvolvido um modelo classificador preditivo da carga de trabalho de enfermagem, utilizando IA. O resultado do modelo demonstrou que é possível treinar algoritmos com dados do PEP para predizer a carga de trabalho de enfermagem. As variáveis tempo de internação, realizar banho de leito, risco de queda e implementar protocolo de prevenção e tratamento de feridas representaram a maior carga de trabalho na classificação final do modelo, dando visibilidade a aspectos relevantes a serem considerados no planejamento das escalas de atividades da equipe de enfermagem e do dimensionamento de pessoal no contexto estudado. O desenvolvimento de modelos classificadores preditivos da carga de trabalho de enfermagem implica na possibilidade de automatizar a atividade, tornando-a objetiva, precisa e proporcionando que os enfermeiros possam destinar o tempo dedicado à avaliação para outras demandas assistenciais e gerenciais. O modelo preditivo tem potencial para ser aplicado em diferentes ambientes, desde que os algoritmos sejam treinados com conjuntos de dados qualificados e representem a realidade de cada local.
